# High and Highly Variable Spontaneous Mutation Rates in *Daphnia*

**DOI:** 10.1093/molbev/msaa142

**Published:** 2020-06-10

**Authors:** Eddie K H Ho, Fenner Macrae, Leigh C Latta, Peter McIlroy, Dieter Ebert, Peter D Fields, Maia J Benner, Sarah Schaack

**Affiliations:** 1 Department of Biology, Reed College, Portland, OR; 2 Division of Natural Sciences and Mathematics, Lewis-Clark State College, Lewiston, ID; 3 Department of Environmental Sciences, Zoology, University of Basel, Basel, Switzerland

**Keywords:** base substitution, gene conversion, heterozygosity, mutation spectrum, evolution, mutation accumulation, Cladocera, Crustacea

## Abstract

The rate and spectrum of spontaneous mutations are critical parameters in basic and applied biology because they dictate the pace and character of genetic variation introduced into populations, which is a prerequisite for evolution. We use a mutation–accumulation approach to estimate mutation parameters from whole-genome sequence data from multiple genotypes from multiple populations of *Daphnia magna*, an ecological and evolutionary model system. We report extremely high base substitution mutation rates (${\overset{-}{µ}}_{n,\mathrm{bs}}$ = 8.96 × 10^−9^/bp/generation [95% CI: 6.66–11.97 × 10^−9^/bp/generation] in the nuclear genome and ${\overset{-}{µ}}_{m,\mathrm{bs}}$ = 8.7 × 10^−7^/bp/generation [95% CI: 4.40–15.12 × 10^−7^/bp/generation] in the mtDNA), the highest of any eukaryote examined using this approach. Levels of intraspecific variation based on the range of estimates from the nine genotypes collected from three populations (Finland, Germany, and Israel) span 1 and 3 orders of magnitude, respectively, resulting in up to a ∼300-fold difference in rates among genomic partitions within the same lineage. In contrast, mutation spectra exhibit very consistent patterns across genotypes and populations, suggesting the mechanisms underlying the mutational process may be similar, even when the rates at which they occur differ. We discuss the implications of high levels of intraspecific variation in rates, the importance of estimating gene conversion rates using a mutation–accumulation approach, and the interacting factors influencing the evolution of mutation parameters. Our findings deepen our knowledge about mutation and provide both challenges to and support for current theories aimed at explaining the evolution of the mutation rate, as a trait, across taxa.

## Introduction

Mutation rates are among the most important parameters in biology, as they are critical for understanding how genetic variation is generated (Lynch et al. 2016). Genetic variation, in turn, provides both the fodder necessary for beneficial evolutionary changes (e.g., adaptation) and mutational inputs that can have deleterious effects (e.g., the accrual of high genetic loads). Despite their importance, direct estimates of mutation rates are a major challenge and not widely available for most eukaryotes (Halligan and Keightley 2009). Mutation–accumulation (MA) experiments (which minimize selection), combined with high-throughput sequencing (which maximize genomic sampling), have made accurate estimates of mutation rates more feasible (Katju and Bergthorsson 2019).

Because rates have, historically, been challenging to measure (Baer et al. 2007), estimates of base substitution rates from a single or few genotypes have often provided the first glimpse of the mutation parameters for a species and have been assumed to reflect the rate for all types of mutation. There are many factors, however, that determine rates of mutation and DNA repair among different types of mutation (e.g., base substitutions vs. transposable element insertions), so the estimates for all rate types may not correlate. Recent evidence also suggests that there may be more intraspecific variation in mutation parameters than previously appreciated (e.g., Narasimhan et al. 2017; Sasani et al. 2019), making it difficult to extrapolate from one or two genotypes to an entire species.

Although differences in mutation parameters among species with major differences in physiology, life history, and/or effective population size are predicted by population genetics and evolutionary theory (Lynch et al. 2016), understanding “how” the mutation rate evolves, as a trait, requires a thorough investigation of the variation at the population level. Here, we estimate base substitution mutation rates, gene conversion rates, and the spectrum of mutation for nine genotypes of *Daphnia magna* collected from three populations along a latitudinal gradient and compare these with other multicellular eukaryotes for which such estimates exist. Examining the mean values, ranges, and correlations among mutation parameters at four levels (among line, genotype, population, and species) using an MA approach reveals variation in the mutational landscape. Such variation determines the potential not only for evolution (i.e., heritability and evolvability) of the mutation rate, as a trait, but also for evolutionary change in all traits. Finally, by comparing the patterns of mutation measured in the lab with the long-term variation expected and observed genomewide, we can determine where mutation and other evolutionary forces, such as selection, intersect to shape genetic variation in nature.

To calculate and compare mutation rates intra- and interspecifically, we sequenced the whole genome of MA lines propagated from three ancestral genotypes originating from three populations (Finland, Germany, and Israel) of *D. magna* (*n *=* *9 genotypes and *n *=* *66 MA lines), used publicly available sequence data from MA lines from the congener *D. pulex* (Keith et al. 2016), and collected rates from the literature for all other animals for which direct estimates are available for both nuclear and mitochondrial genomes (see supplementary Materials and Methods and fig. S1, Supplementary Material online).

## Results

**Fig. 1:**
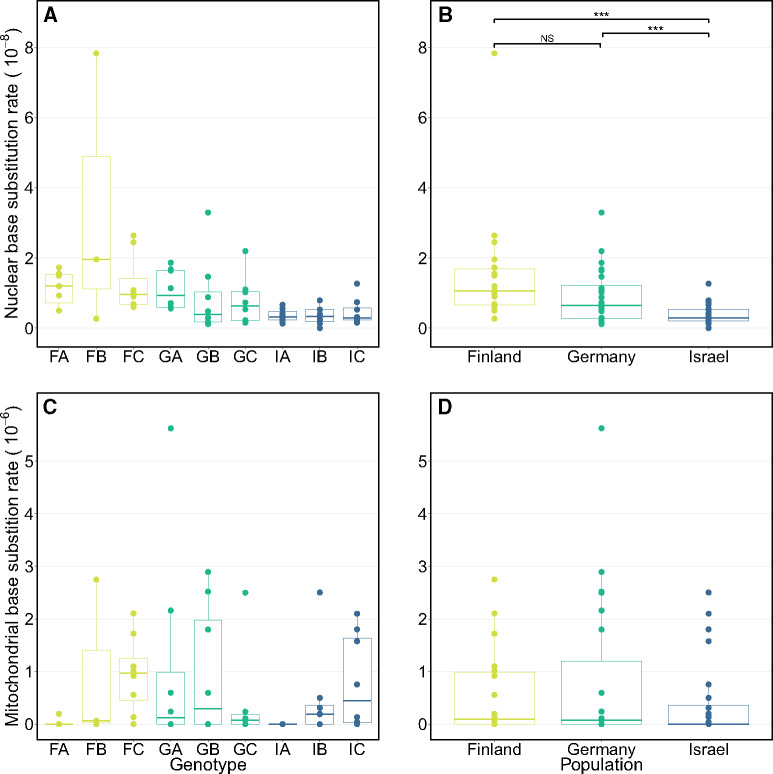
Box plots of base substitution mutation rates (per site per generation) for the nuclear (*A* and *B*; top) and mitochondrial (*C* and *D*; bottom) genomes for each genotype (left) and population (right) based on whole genome sequencing of 66 D. magna MA lines derived from nine genotypes collected in Finland (FA, FB, FC), Germany (GA, GB, GC), and Israel (IA, IB, IC). Results of post-hoc Tukey tests are indicated with brackets ‘NS’ and ‘***’ represents *P*-values > 0.05 and < 0.05, respectively. Note: The mitochondrial base substitution rate for one MA line, GC8, was an order of magnitude higher than other MA lines and was excluded from plots (*C*, *D*) for clarity but is reported in supplementary table S1*B*, Supplementary Material online.

*Daphnia magna* exhibit the highest and most variable nuclear base substitution mutation rates observed in eukaryotes using an MA approach (${\overset{-}{µ}}_{n,\mathrm{bs}}$= 8.96 × 10^−9^/bp/generation [95% CI: 6.66–11.97 × 10^−9^/bp/generation]), with rates spanning an order of magnitude among genotypes (3.6 × 10^−9^ to 3.4 × 10^−8^/bp/generation; fig. 1*A*) and varying among populations (fig. 1*B*;  *F*_2,6_ = 20.36, *P *=* *0.0021; supplementary tables S1*A* and S1*B* and Results, Supplementary Material online). In the mtDNA, the variation among genotypes is even greater (spanning >3 orders of magnitude; fig. 1*C* and supplementary table S1*A*, Supplementary Material online) and the mean base substitution mutation rate is an order of magnitude higher than the previous highest mitochondrial mutation rate reported in animals using an MA approach (${\overset{-}{µ}}_{m,\mathrm{bs}}$ = 8.7 × 10^−7^/bp/generation; [95% CI: 4.40–15.12 × 10^−7^/bp/generation] in *D. magna* vs. 9.7 × 10^−8^/bp/generation in *Caenorhabditis elegans* (Denver 2000)). Although the rates of mutation in both the nuclear and the mitochondrial genome of *D. magna* are very high, rates do not covary across genotypes or populations between these two genomes (e.g., Finnish genotypes have the highest nuclear rates, but not the highest mtDNA rates; ρ = –0.02, *t*_64_ = –0.18, *P *=* *0.85; fig. 1*B* and *D* and supplementary table S10, Supplementary Material online), and in fact, the ratio of the mtDNA rate to the nuclear rate varies by two orders of magnitude (from 0 to 331; supplementary table S1*A*, Supplementary Material online). The different subcellular environments, DNA polymerases, replication rates, and mechanisms of local DNA damage/repair in the nuclear and mitochondrial genome likely explain the lack of covariation in their mutation rates, but the consequences of different ratios of rates could be significant. In the short term, different rate ratios can impact the coevolution of interacting mtDNA- and nuclear-encoded proteins operating in the mitochondrial matrix (Rand et al. 2004). Over long periods, mtDNA rates have diverged to puzzling extremes (e.g., in plants, they are typically very low, whereas in animals, they are typically very high, relative to the nuclear rates; Havird and Sloan 2016).

**Fig. 2:**
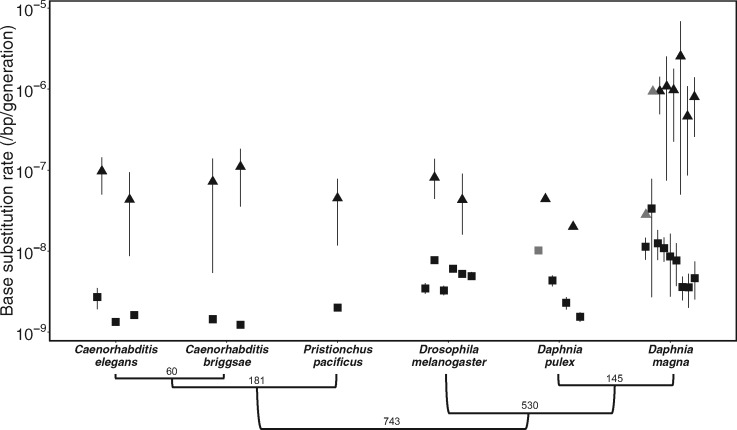
Nuclear (squares) and mitochondrial (triangles) base substitution mutation rates (per site per generation) for each genotype measured from all six eukaryotic species for which direct rate estimates for both genomes are available based on sequenced MA lines (see Supplementary References [3, 7, 8, 15, 24, 33, 54, 55, 57-66], Supplementary Material online). Error bars represent the 95% CI estimated from bootstrapping (when reported) or extrapolating the SE, depending on the study; grey points lack error bars because the lower 95% CI overlaps zero. Below the x axis, divergence times (Ma) and branching topology are illustrated for all six species based on TimeTree estimates (67).

To investigate variation in base substitution mutation rates between species, we compared our estimates with all other animals for which MA-derived data or estimates for nuclear and mitochondrial base substitution mutation rates exist, including the congener, *D. pulex* (fig. 2 and supplementary Results, Supplementary Material online). The base substitution mutation rates in *D. magna* (both nuclear and mitochondrial) are among the highest observed in animals, including *D. pulex* that has few major differences in morphology, behavior, physiology, and life history (fig. 2). Mean µ_*n*,__bs_ in *D. magna* was 1.95 times higher than that of *D. pulex* (4.59 × 10^−9^/bp/generation), consistent with previously observed patterns for microsatellite mutation rates, which were an order of magnitude greater in *D. magna* than in *D. pulex* (Flynn et al. 2017; Ho et al. 2019). In both species, the estimates of µ_*n*,__bs_ span an order of magnitude (*D. magna*: 3.57 × 10^−8^–3.35 × 10^−8^/bp/generation and *D. pulex*: 1.55 × 10^−9^–1.02 × 10^−8^/bp/generation), which is greater than the intraspecific variation observed in other animals but is similar to that observed in unicellular eukaryotes, such as *Chlamydomonas reinhardtii* (Ness et al. 2015) and *Saccharomyces cerevisiae* (Gou et al. 2019).

Explanations for the evolution of the mutation rate among lineages are numerous but can be broadly categorized into three main classes: proximal, life history-related, and population genetic. Proximal mechanisms (e.g., genetic “quality,” body temperature, and/or metabolic rate determine mutation rates) have been implicated in a number of studies (Sharp and Agrawal 2016), but causality among many of the potential factors is difficult to determine because many traits are confounded. Life history-related theories which argue, e.g., that age-at-maturity or parental age dictate the number of rounds of DNA replication prior to gametogenesis and thus determine mutation rates (Kong et al. 2012; Latta et al. 2013; Thomas et al. 2018; Gou et al. 2019) have empirical support in some species but have not been broadly tested. With regards to our *D. magna* populations, fitness-related traits (such as body size and egg number) and life history parameters (e.g., age-to-maturity) do not differ substantially among the three populations assayed and do not explain the intraspecific variation in base substitution rates (supplementary fig. S2, Supplementary Material online).

Broader explanations for mutation rate variation among species based on population genetics posit that the ability of natural selection to, for example, favor high fidelity polymerases or purge mutations that lower mutation rates is overwhelmed by the power of genetic drift to fix alleles in small populations (Lynch et al. 2016). Referred to as the “drift-barrier” hypothesis, this theory has two predictions as follows: 1) there is a negative correlation between mutation rates and effective population size across species and 2) species with similar effective population sizes will have similar mutation rates. Although the drift-barrier hypothesis does not make explicit predictions about levels of intraspecific variation, it implicitly suggests that interspecific variation among lineages with major differences in their population genetic parameters would eclipse the differences among genotypes or populations of the same species.

To see if these data are consistent with the drift-barrier hypothesis, we estimate the effective population size (*N*_e_) of *D. magna* and *D. pulex* using the reported genetic diversity at synonymous sites (π_s_) from Haag et al. (2009) and the mean µ_*n*__,bs_ of each species to solve for *N*_e_ (using the equation π* *= 4 *N*_e_ µ). The estimated *N*_e_ for *D. magna* and *D. pulex* are ∼418,000 and 1,198,000, respectively, which is consistent with the drift-barrier hypothesis predictions (i.e., that populations with larger *N*_e_ exhibit lower mutation rates). However, as estimates for both species span an order of magnitude, we must use caution when calculating the mean µ_*n*,__bs_ “of the species.” For example, if mutation rates from Israel genotypes are actually more representative for the species, in general, then *D. magna* would have a lower µ_*n*__,bs_ (3.91 × 10^−9^/bp/generation) and a lower *N*_e_ (954,000) compared with *D. pulex*. This is a potential problem for testing the drift-barrier hypothesis for any species with significant intraspecific variation.

In addition to directly estimating mutation rates, we calculated the heritability (the proportion of the phenotypic variation explained by genetic variation) and evolvability (the per generation input of mutational variance) for the mutation rate, as a trait, in *D. magna* (0.014 and 0.016, respectively; supplementary table S2, Supplementary Material online). Although the calculated values may seem low, most previous estimates have been zero (e.g., in fly, Fernández and López-Fanjul 1996 and *D. magna*, Eberle et al. 2018). Although it is well known that intraspecific variation in traits is a prerequisite for the evolution of the trait, it is especially interesting to obtain a glimpse of the genetic component of the variation and the mutational variance that can be introduced into this trait, in particular. It may be tempting to think of the mutation rate as a static trait that, although affecting the rate of evolution of other traits, itself is somehow set or immovable, but this is not the case. Like any other trait, understanding the evolution of the mutation rate depends on knowing the intraspecific variation in the trait, its heritability, and propensity to evolve.

Whereas mutations introduce genetic variation and increase heterozygosity in the genome, gene conversion is a process that reduces heterozygosity over time (homolog-dependent DNA repair either renders a mutation homozygous or leads to the loss of the mutation, Chen et al. 2007). The importance of gene conversion in shaping the fate of new mutations and genome content over time is often overlooked (but see Daugherty and Zanders 2019) and events are challenging to detect, thus direct estimates of gene conversion rates are even more scarce than mutation rates (but see Keith et al. 2016; Flynn et al. 2017; Sharp and Agrawal 2018). Gene conversion events are important, however, because they can 1) result in DNA changes at multiple sites with a single event (i.e., gene conversion “tracts”) and/or 2) unmask the recessive effects of new mutations by making a locus homozygous even without sexual reproduction. We estimated gene conversion rates based on 35 observed sites distributed among 13 gene conversion tracts yielding a mean gene conversion rate (µ_*n*,__g_) of 6.13 × 10^−7^ per heterozygous site/generation, and an order of magnitude higher rates than those observed in the large population controls, suggesting gene conversion events, like mutations, are deleterious (see supplementary Results, Supplementary Material online). Although the mean rate is intermediate compared with previous reports in *D. pulex* (Omilian et al. 2006; Keith et al. 2016; Flynn et al. 2017), it is difficult to compare rates across studies because the methods for calculating rates are not standardized. Within our experiment, however, the intraspecific variation in gene conversion rates reported is unprecedented (ranging from 0 to 55.8 × 10^−5^ per heterozygous site/generation; supplementary table S8*A*, Supplementary Material online), which could have profound effects on levels of heterozygosity.

Although mutations should lead to an increase in heterozygosity over time, in theory, in our study, µ_*n*__,bs_ is not correlated with heterozygosity across genotypes (ρ = –0.43, *t*_7_ = –1.26, *P *=* *0.25; supplementary table S9*A* and fig. S4, Supplementary Material online). The lack of a positive correlation between heterozygosity and µ_*n*__,bs_ could be explained by several other factors which can affect levels of heterozygosity as well. First, if gene conversion is the major factor eroding genetic diversity, the expected heterozygosity at equilibrium can be estimated as µ_*n*__,bs_/(µ_*n*__,bs_ + µ_*n*__,g_). In this scenario, based on our data, IA would have the highest expected level of heterozygosity despite possessing the lowest base substitution mutation rate (supplementary table S9*B*, Supplementary Material online). For those starting genotypes where we observed both base substitution mutations and gene conversion events, we calculated expected and observed heterozygosity (supplementary table S9*B*, Supplementary Material online) and found no relationship, supporting the notion that although mutation is the ultimate source of genetic variation, it is not the only factor determining levels of genetic diversity. In particular, because the historical effective population size (*N*_e_) experienced by genotypes prior to the experiment would dictate the relative importance of genetic drift in determining levels of neutral diversity within a population. Factors influencing *N*_e_ that could be especially important in *D. magna* are 1) the frequency of sex (*D. magna* are cyclical parthenogens and thus can reproduce asexually or sexually), 2) levels of inbreeding, 3) climatic factors, such as temperature and humidity, that can lead to habitat size fluctuations, and 4) variable rates of recombination that can affect the strength of linked selection (Ellegren and Galter 2016). Differences in these genetic and ecological factors between the *D. magna* genotypes and populations we sampled could interfere with a positive correlation between µ_*n*__,bs_ and heterozygosity. In fact, the population samples here are intended to represent a latitudinal gradient and thus a range of abiotic conditions. Finnish populations likely experience more frequent bottlenecks than either Israeli or German populations, because they experience both freezing temperatures in the winter and complete dry downs in the summer (Lange et al. 2015). In contrast, the more southerly populations only exhibit one or the other (i.e., German populations experience freezing temperatures but do not dry down, whereas Israeli populations dry down but never freeze).

**Fig. 3:**
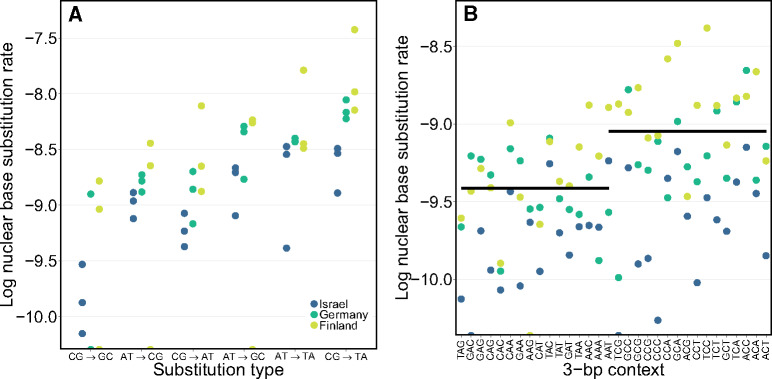
Mean conditional base substitution rates (per site per generation) for (*A*) each possible type of substitution for each genotype and (*B*) each of the 32 possible 3-bp local contexts for each population. The black bar on the left and right of the plot indicates the conditional rate averaged across all populations across the contexts with a central ‘A’ and central ‘C’, respectively. Dots represent genotypes (top) and population (bottom) means from Finland (yellow), Germany (green), and Israel (dark blue), respectively.

Although rates exhibit high levels of intraspecific variation, the spectrum of mutation does not vary greatly among genotypes and populations (supplementary tables S3*A* and S4, Supplementary Material online). Instead, the spectrum varies based on the substitution type, central nucleotide, and local genomic context (fig. 3). We used the frequency of all six possible base substitution types (supplementary table S3*A*, Supplementary Material online) to calculate the base substitution mutation rates for each type of mutation (fig. 3*A*) and for each nucleotide (C or G vs. A or T) in all possible 3-bp contexts (fig. 3*B*). As expected, we find rates vary among different substitution types in *D. magna* (χ^2^ = 337.7, df = 5, *P *<* *0.001), although surprisingly the frequency of different types differs from the pattern in *D. pulex* (supplementary table S3*B*, Supplementary Material online). The most common mutation type (CG → TA) is the same in both species, however, and the mean Ts:Tv ratios are well within the range reported in invertebrates so far (1.54 [nucDNA] and 2.86 [mtDNA]; supplementary table S3*C*, Supplementary Material online). Generally, base substitution mutation rates in *D. magna* are 1.9× higher when the center nucleotide is C (or G) compared with when it is A (or T) (*F*_1,232_ = 25.6, *P *<* *0.0001; fig. 3*B*) and local context (neighboring base pairs) affects these rates (*F*_15,232_ = 3.6, *P *<* *0.0001; supplementary table S4, Supplementary Material online). Specifically, when the center nucleotide is C (or G), rates are lower at CpG sites than non-CpG sites (supplementary table S5, Supplementary Material online; *t_7_* = 5.29, *p *=* *0.0011).

The consistency of the differences based on spectrum suggests that there are distinctive mechanisms governing particular changes, but that those mechanisms operate across genotypes, populations, and (to some degree) species. Because we were able to observe mutation rates and spectra in an MA framework, where selection is minimized, we can use those parameters to estimate expected GC content genomewide at equilibrium (supplementary table S6, Supplementary Material online). We can compare those estimates to observed GC content (based on the whole-genome sequence of the ancestors of each genotype used in the experiment). Predicted equilibrium GC content for each genotype ranges from 31% to 66%, whereas observed GC content is consistently ∼41% (supplementary fig. S3, Supplementary Material online). This pattern suggests natural selection, not mutation, is the primary force shaping GC content genomewide.

**Fig. 4:**
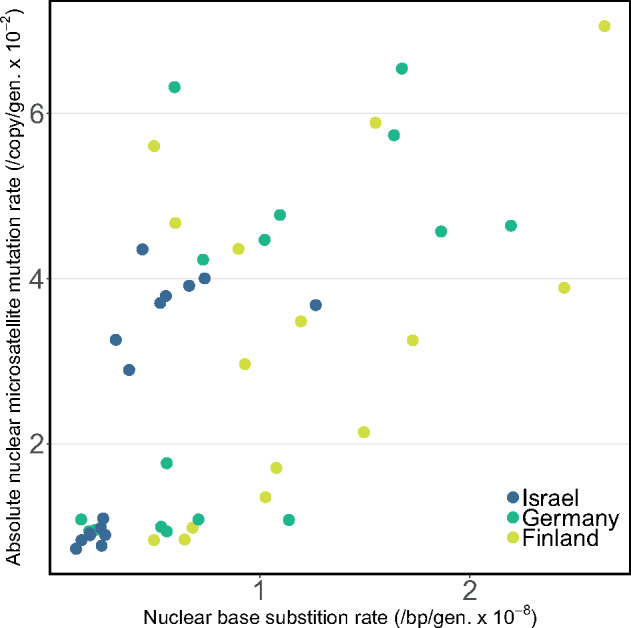
Correlated (*ρ* = 0.61, *t*_45_ = 5.1, p < 0.0001) mean nuclear base substitution rates (per bp per generation) and mean absolute nuclear microsatellite mutation rates (per copy per generation) from a subset of MA lines for which both estimates are available (FA and FC from Finland (in yellow), GA and GC from Germany (in green), IA and IC from Israel (in dark blue); see Ho et al. 2019).

The extent to which rates of different types of mutations correlate within a lineage remains largely unknown. Here, we examine the correlation between nuclear base substitution mutation rates and microsatellite mutation rates (µ_*n*__,ms_) for a subset of the same *D. magna* MA lines analyzed in a previous study (supplementary Materials and Methods, Supplementary Material online, Ho et al. 2019). Microsatellite loci are repetitive regions of the genome known for their propensity to mutate rapidly. Although population genetics theory would predict a positive correlation among rates regardless of mutation type, the molecular mechanisms that cause microsatellite loci to mutate, such as retrotransposition, unequal crossing over, and DNA slippage (Ellegren 2004), are largely distinct from those known to cause base substitutions. To test this idea, we looked for correlations between µ_*n*__,bs_ and the absolute value of mutation rates at microsatellite loci (|µ_*n*__,ms_|), because they can either increase or decrease in size. We observe a positive correlation between µ_*n*__,bs_ and |µ_*n*__, ms_| (fig. 4 and supplementary table S10, Supplementary Material online; ρ = 0.61, *t*_45_ = 5.1, *P *<* *0.0001), which is consistent with a strong role of the population genetic environment in determining the evolution of mutation rates.

## Discussion

Mutations provide the ultimate source of genetic variation and, as such, understanding the rate and spectrum of mutation, as well as rates of gene conversion, is of key importance in biology. As the debates about the primacy of natural selection or genetic drift as evolutionary influences wage on (Kern and Hahn 2018), characterizing the mutational landscape provides essential baseline knowledge required for understanding how genetic variation is generated and shaped in nature (Sniegowski et al. 2000). Here, we report direct estimates of the rate of mutation for base substitutions and are able to compare data among lines, genotypes, populations, and species to gain insights into both the intraspecific variation in mutation rates and their heritability and evolvability. Further, we use nucleotide variants to estimate gene conversion rates, the spectrum of mutation, and expected versus observed levels of heterozygosity. Last, we compare rate estimates for base substitutions to recently reported estimates of microsatellite mutation rates in order to understand if the high mutation rates observed are a general feature, rather than exclusive to one mutation type.

The MA-derived direct estimates of base substitution mutation rates we report for *D. magna* are the highest rates and exhibit the greatest levels of variation in base substitution mutations rates among animals for which such experimental data exist (fig. 1). Notably, the rate estimates are higher than those published for the congener, *D. pulex*, an organism with which *D. magna* shares numerous ecological, physiological, morphological, and life history–based similarities. It is important to note that rate estimates from different studies, even those made using an MA approach and using whole-genome sequencing, can be sensitive to differences in depth of coverage or the quality/availability of a reference genome, so comparisons across studies must be interpreted with some caution. In addition, for many species, MA lines are not ethical or feasible, such as humans and chimps, but our highest genotype-specific rate estimates are also higher than the mean rate estimates for these species (1.29 × 10^−8^ mutations per base per meiosis in humans, Tian et al. 2019 and 1.48 × 10^−8^ per base per generation in chimps, Tatsumoto et al. 2017). Although increasing the range of known mutation rates is important for understanding the accrual of deleterious mutation loads, the risk of genetic disease, the rate at which genetic variation is introduced into populations, and the high levels of intraspecific variation in mutation rates, it is also significant for deepening our understanding of the evolution of the mutation rate.

The nuclear rates estimated across nine genotypes sampled from three populations spanned an order of magnitude, whereas the mtDNA base substitutions mutation rates spanned three orders of magnitude (table 1). The intraspecific variation observed, as well as the evidence for nonzero levels of heritability of the mutation rate as a trait, highlights the potential for rapid evolution of the mutation rate among populations and species and underscores the need for more multigenotype, multipopulation direct estimates of mutation parameters. Notably, nuclear and mtDNA rates do not covary across genotypes (supplementary table S10, Supplementary Material online) and, in fact, exhibit a range of ratios of µ_*m*__,bs_:µ_*n*__,bs_ from 0 to over 300 (supplementary table S1*A*, Supplementary Material online). Proximally, this enormous span has implications for mitochondrial function (as that depends on the interaction of mitochondrially and nuclear-encoded proteins) and thus fitness. Ultimately, such a discordance in rates among genomic partitions could impact the strength of the selective pressure favoring a switch to sexual reproduction or outcrossing, as posited by the mitonuclear sex hypothesis (Havird et al. 2015).

**Table 1.** Base Substitution Mutation Rates per bp/Generation, per Genome/Generation (for the nuclear genome), and per bp/Day for Both the Nuclear and Mitochondrial Genome of *Daphnia magna* by Genotype, Population (F, Finland; G, Germany; I, Israel), and Specieswide.

		Nuclear Rates			mtDNA Rates	
		**Per bp/Generation (10** ^-^ **^9^)**	Per Genome/Generation	Per bp/Day (10^-10^)	**Per bp/Generation (10** ^-^ **^7^)**	Per bp/Day (10^-8^)
Genotype	FA	11.28	2.69	7.18	0.28	0.19
	FB	33.53	7.98	17.71	9.37	5.34
	FC	12.53	2.98	6.99	9.43	5.48
	GA	10.93	2.60	6.75	10.77	7.32
	GB	8.57	2.04	5.39	9.76	6.67
	GC	7.68	1.83	4.84	25.46	17.20
	IA	3.60	0.86	2.08	0.00	0.00
	IB	3.57	0.85	2.14	4.63	2.69
	IC	4.63	1.10	2.78	8.01	4.54
Population	F	15.54	3.70	8.85	5.86	3.40
	G	9.06	2.16	5.66	15.33	10.40
	I	3.93	0.94	2.33	4.21	2.41
Species	*D. magna*	8.96	2.13	5.32	8.70	5.59

In addition to estimating base substitution mutation rates, we used this data set to estimate gene conversion rates—an important and underinvestigated parameter in evolutionary biology the phenotypic impacts of which have rarely been discussed. Much like the estimate of the mutation rate, gene conversion rates ranged widely among genotypes based on a very stringent set of filter steps that likely provide a lower bound estimate for the frequency of these events. Interestingly, gene conversion rate estimates from the MA lines were 10× higher than in the large population control lineages, suggesting that the minimization of selection via single progeny propagation allows mutations not only to accumulate but also gene conversion events to occur unabated by natural selection.

Estimating rates of gene conversion is important, given the role this process plays in erasing heterozygosity introduced by mutation (either by eliminating or fixing the new allele). Indeed, our estimates of observed heterozygosity revealed 1) populations with the highest mutation rates do not have the highest levels of heterozygosity (supplementary table S9*A*, Supplementary Material online) and 2) a lack of concordance with expected levels (supplementary table S9*B*, Supplementary Material online). The explanation for the former could be 1) intraspecific variation in gene conversion rates or 2) differences in the historical *N*_e_ among the populations these genotypes were sampled from which could potentially be quite dramatic given seasonal fluctuations in temperature and humidity experienced in Finland, Germany, and Israel.

Using the mutation rate estimates for *D. magna* and *D. pulex*, we were able to calculate *N*_e_ for each species to assess whether, specieswide, the difference in rate estimates observed are consistent with predictions of the drift-barrier hypothesis (Lynch et al. 2016). Overall, the *N*_e_ of *D. magna* is much lower (about one-third) than *D. pulex*, which is predicted to correspond to a higher average mutation rate, which we observe (fig. 2). This begs the question, of course, whether the rate estimates at the higher or lower end of the range we report are, in fact, more representative of the species as a whole if one were able to sample even more genotypes.

Although the intraspecific variation in rates among *D. magna* was remarkable, perhaps as striking was the consistency of the spectrum of mutation regardless of rate (fig. 3). This constancy suggests that, although evolutionary forces can act to shape the mutation rate and either drive or permit it to be relatively high or low, the molecular mechanisms underlying the mutations themselves may be highly constrained.

Although high rates alone require us to reconsider the pace at which evolutionary phenomena (such as adaptation and extinction) can occur in different taxa, the unexpected levels of intraspecific variation observed expand the known parameter space upon which evolutionary forces can act to shape the mutation rate, itself, as a trait. Although our findings challenge the notion that any one of the current slate of theories aimed at identifying the factors governing mutation rate evolution can provide the sole explanation for how these fundamental parameters in biology vary and evolve, the positive correlation between base substitution and microsatellite rates (fig. 4) supports a significant role for the population genetic environment. Given the high levels of intraspecific variation in mutation rates reported here, future studies should continue to incorporate multiple genotypes and populations in estimates of mutation parameters in order to better understand the causes and the consequences of such variation.

## Materials and Methods

For detailed methods, see supplementary Materials and Methods, Supplementary Material online. Individual female descendants from each of the nine ancestral genotypes (originally collected from Finland, Germany, and Israel) were used to establish MA lines (*n *=* *66 total; see supplementary fig. S1 for study design, Supplementary Material online). Each MA line was propagated from generation to generation via single offspring descent by taking a single neonate from the second clutch and transferring it to a new beaker. Immediate descendants of the individuals used to initiate the lines were both 1) preserved for later sequencing (“starting controls”) and 2) used to initiate large population controls to run in parallel with the MA lines (“extant controls”) to be sequenced at the end of the MA period. Five clonal individuals from each line and control were flash frozen for DNA extractions. DNA was extracted and 94 Wafergen DNA 150 bp paired-end libraries were prepared using the Biosystems Apollo 324 NGS library prep system. Libraries were pooled and sequenced on an Illumina Hiseq 3000. Reads were processed, trimmed, and mapped to assembled genomes to call variants (supplementary table S11, Supplementary Material online). A subset of observed events was validated with Sanger sequencing to established filter thresholds to estimate rates and spectra (supplementary table S12, Supplementary Material online). The nuclear base substitution mutation rate of each MA line was calculated as follows: µ_*n*__,bs_ = *x*_bs_/(*g* × 2*n*), where *x*_bs_ represents the number of base substitution mutations that passed the above filters, *g* represents the number of MA generations, and *n* number of callable sites (2*n* represents the number of diploid bases). The mitochondrial base substitution rate was calculated assuming neutrality (Haag-Liautard et al. 2008) as µ_*m*__,bs_ = ∑*i f_i_*/(*g* × *n*), where *f_i_* is the allele frequency for mutation *i*, *g* is the number of MA generations, and *n* is the length of the mitochondrial genome. The gene conversion rate for each MA line was calculated as µ_*n*__,g_ = *x*_g_/(*g* × *n*), where *x*_g_ represents the number of gene conversion sites, *g* represents the number of MA generations, and *n* represents the number of heterozygous sites in the ancestral genotype.

Additional information on sequencing protocols, assembly, variant calling, rate estimation, spectrum analysis, validation procedures, and statistical tests are detailed in the supplementary Materials and Methods, Supplementary Material online.

## Supplementary Material

Supplementary data are available at *Molecular Biology and Evolution* online.

## Supplementary Material

msaa142_Supplementary_Data

## Notes

The *Daphnia magna* reference genome used is part of BioProject ID PRJNA624896.

## References

[msaa142-B1] Baer CF , Miyamoto MM, Denver DR. 2007. Mutation rate variation in multicellular eukaryotes: causes and consequences. Nat Rev Genet. 8(8):619–631. 17637734 10.1038/nrg2158

[msaa142-B2] Chen JM , Cooper DN, Chuzhanova N, Férec C, Patrinos GP. 2007. Gene conversion: mechanisms, evolution and human disease. Nat Rev Genet. 8(10):762–775. 17846636 10.1038/nrg2193

[msaa142-B3] Daugherty MD , Zanders SE. 2019. Gene conversion generates evolutionary novelty that fuels genetic conflicts. Curr Opin Genet Dev. 58–59:49–54. 10.1016/j.gde.2019.07.011 PMC6889005 31466040

[msaa142-B4] Denver DR. 2000. High direct estimate of the mutation rate in the mitochondrial genome of *Caenorhabditis elegans*. Science 289(5488):2342–2344. 11009418 10.1126/science.289.5488.2342

[msaa142-B5] Eberle S , Dezoumbe D, McGregor R, Kinzer S, Raver W, Schaack S, Latta LC. 2018. Hierarchical assessment of mutation properties in *Daphnia magna*. G3 8:3481–3487. 30158321 10.1534/g3.118.200472 PMC6222573

[msaa142-B6] Ellegren H. 2004. Microsatellites: simple sequences with complex evolution. Nat Rev Genet. 5(6):435–445. 15153996 10.1038/nrg1348

[msaa142-B7] Ellegren H , Galtier N. 2016. Determinants of genetic diversity. Nat Rev Genet. 17(7):422–433. 27265362 10.1038/nrg.2016.58

[msaa142-B8] Fernández J , López-Fanjul C. 1996. Spontaneous mutational variances and covariances for fitness-related traits in *Drosophila melanogaster*. Genetics 143(2):829–837. 8725231 10.1093/genetics/143.2.829 PMC1207341

[msaa142-B9] Flynn JM , Caldas I, Cristescu ME, Clark AG. 2017. Selection constrains high rates of tandem repetitive DNA mutation in *Daphnia pulex*. Genetics 207(2):697–710. 28811387 10.1534/genetics.117.300146 PMC5629333

[msaa142-B10] Flynn JM , Chain FJJ, Schoen DJ, Cristescu ME. 2017. Spontaneous mutation accumulation in *Daphnia pulex* in selection-free vs. competitive environments. Mol Biol Evol. 34(1):160–173. 27777284 10.1093/molbev/msw234

[msaa142-B11] Gou L , Bloom JS, Kruglyak L. 2019. The genetic basis of mutation rate variation in yeast. Genetics 211(2):731–740. 30504363 10.1534/genetics.118.301609 PMC6366923

[msaa142-B12] Haag CR , McTaggart SJ, Didier A, Little TJ, Charlesworth D. 2009. Nucleotide polymorphism and within-gene recombination in *Daphnia magna* and *D. pulex*, two cyclical parthenogens. Genetics 182(1):313–323. 19299338 10.1534/genetics.109.101147 PMC2674827

[msaa142-B13] Haag-Liautard C , Coffey N, Houle D, Lynch M, Charlesworth B, Keightley PD. 2008. Direct estimation of the mitochondrial DNA mutation rate in *Drosophila melanogaster*. PLoS Biol. 6(8):e204. 18715119 10.1371/journal.pbio.0060204 PMC2517619

[msaa142-B14] Halligan DL , Keightley PD. 2009. Spontaneous mutation accumulation studies in evolutionary genetics. Annu Rev Ecol Evol Syst. 40(1):151–172.

[msaa142-B15] Havird JC , Hall MD, Dowling DK. 2015. The evolution of sex: a new hypothesis based on mitochondrial mutational erosion: mitochondrial mutational erosion in ancestral eukaryotes would favor the evolution of sex, harnessing nuclear recombination to optimize compensatory nuclear coadaptation. BioEssays 37(9):951–958. 26201475 10.1002/bies.201500057 PMC4652589

[msaa142-B16] Havird JC , Sloan DB. 2016. The roles of mutation, selection, and expression in determining relative rates of evolution in mitochondrial versus nuclear genomes. Mol Biol Evol. 33(12):3042–3053. 27563053 10.1093/molbev/msw185 PMC5100045

[msaa142-B17] Ho EKH , Macrae F, Latta LC, Benner MJ, Sun C, Ebert D, Schaack S. 2019. Intraspecific variation in microsatellite mutation profiles in *Daphnia magna*. Mol Biol Evol. 36(9):1942–1954. 31077327 10.1093/molbev/msz118 PMC6934441

[msaa142-B18] Katju V , Bergthorsson U. 2019. Old trade, new tricks: insights into the spontaneous mutation process from the partnering of classical mutation accumulation experiments with high-throughput genomic approaches. Genome Biol. Evol. 11(1):136–165. 30476040 10.1093/gbe/evy252 PMC6330053

[msaa142-B19] Keith N , Tucker AE, Jackson CE, Sung W, Lucas Lledó JI, Schrider DR, Schaack S, Dudycha JL, Ackerman M, Younge AJ, et al 2016. High mutational rates of large-scale duplication and deletion in *Daphnia pulex*. Genome Res. 26(1):60–69. 26518480 10.1101/gr.191338.115 PMC4691751

[msaa142-B20] Kern AD , Hahn MW. 2018. The neutral theory in light of natural selection. Mol Biol Evol. 35(6):1366–1371. 29722831 10.1093/molbev/msy092 PMC5967545

[msaa142-B21] Kong A , Frigge ML, Masson G, Besenbacher S, Sulem P, Magnusson G, Gudjonsson SA, Sigurdsson A, Jonasdottir A, Jonasdottir A, et al 2012. Rate of de novo mutations and the importance of father’s age to disease risk. Nature 488(7412):471–475. 22914163 10.1038/nature11396 PMC3548427

[msaa142-B22] Lange B , Kaufman AP, Ebert D. 2015. Genetic, ecological and geographic covariables explaining host range and specificity of a microsporidian parasite. J Anim Ecol. 84(6):1711–1719. 26147623 10.1111/1365-2656.12421

[msaa142-B23] Latta LC , Morgan KK, Weaver CS, Allen D, Schaack S, Lynch M. 2013. Genomic background and generation time influence deleterious mutation rates in *Daphnia*. Genetics 193(2):539–544. 23183667 10.1534/genetics.112.146571 PMC3567742

[msaa142-B24] Lynch M , Ackerman MS, Gout J-F, Long H, Sung W, Thomas WK, Foster PL. 2016. Genetic drift, selection and the evolution of the mutation rate. Nat Rev Genet. 17(11):704–714. 27739533 10.1038/nrg.2016.104

[msaa142-B25] Narasimhan VM , Rahbari R, Scally A, Wuster A, Mason D, Xue Y, Wright J, Trembath RC, Maher ER, van Heel DA, et al 2017. Estimating the human mutation rate from autozygous segments reveals population differences in human mutational processes. Nat Commun. 8:303. 28827725 10.1038/s41467-017-00323-y PMC5566399

[msaa142-B26] Ness RW , Morgan AD, Vasanthakrishnan RB, Colegrave N, Keightley PD. 2015. Extensive do novo mutation rate variation between individuals and across the genome of *Chlamydomonas reinhardtii*. Genome Res. 25(11):1739–1749. 26260971 10.1101/gr.191494.115 PMC4617969

[msaa142-B27] Omilian AR , Cristescu MEA, Dudycha JL, Lynch M. 2006. Ameiotic recombination in asexual lineages of Daphnia. Proc Natl Acad Sci U S A. 103(49):18638–18643. 17121990 10.1073/pnas.0606435103 PMC1693715

[msaa142-B28] Rand DM , Haney RA, Fry AJ. 2004. Cytonuclear coevolution: the genomics of cooperation. Trends Ecol Evol. 19(12):645–653. 16701327 10.1016/j.tree.2004.10.003

[msaa142-B29] Sasani TA , Pedersen BS, Gao Z, Baird L, Przeworski M, Jorde LB, Quinlan AR. 2019. Large, three-generation human families reveal post-zygotic mosaicism and variability in germline mutation accumulation. eLife 8:e46922. 31549960 10.7554/eLife.46922 PMC6759356

[msaa142-B30] Sharp NP , Agrawal AF. 2016. Low genetic quality alters key dimensions of the mutational spectrum. PLoS Biol. 14(3):e1002419. 27015430 10.1371/journal.pbio.1002419 PMC4807879

[msaa142-B31] Sniegowski PD , Gerrish PJ, Johnson T, Shaver A. 2000. The evolution of mutation rates: separating causes from consequences. BioEssays 22(12):1057–1066. 11084621 10.1002/1521-1878(200012)22:12<1057::AID-BIES3>3.0.CO;2-W

[msaa142-B32] Tatsumoto S , Go Y, Fukuta K, Noguchi H, Hayakawa T, Tomonaga M, Hirai H, Matsuzawa T, Fujiyama AK. 2017. A. Direct estimation of *de novo* mutation rates in a chimpanzee parent-offspring trio by ultra-deep whole genome sequencing. Sci Rep. 7(1):1–2. 29093469 10.1038/s41598-017-13919-7 PMC5666008

[msaa142-B33] Thomas GWC , Wang RJ, Puri A, Harris RA, Raveendran M, Hughes DST, Murali SC, Williams LE, Doddapaneni H, Muzny DM, et al 2018. Reproductive longevity predicts mutation rates in primates. Curr Biol. 28(19):3193–3197.e5. 30270182 10.1016/j.cub.2018.08.050 PMC6177314

[msaa142-B34] Tian X , Browning BL, Browning SR. 2019. Estimating the genome-wide mutation rate with three-way identity by descent. Am J Hum Genet. 105(5):883–893. 31587867 10.1016/j.ajhg.2019.09.012 PMC6848988

