# Supplementary material for: High and Highly Variable Spontaneous Mutation Rates in Daphnia
Source: Mol Biol Evol. 2020 Jun 10;37(11):3258–66. doi: 10.1093/molbev/msaa142 (PMC7820357; doi:10.1093/molbev/msaa142)
Supplement: msaa142_Supplementary_Data [file msaa142_supplementary_data.zip › msaa142_supplementary_data/SuppFigures052820.pdf]

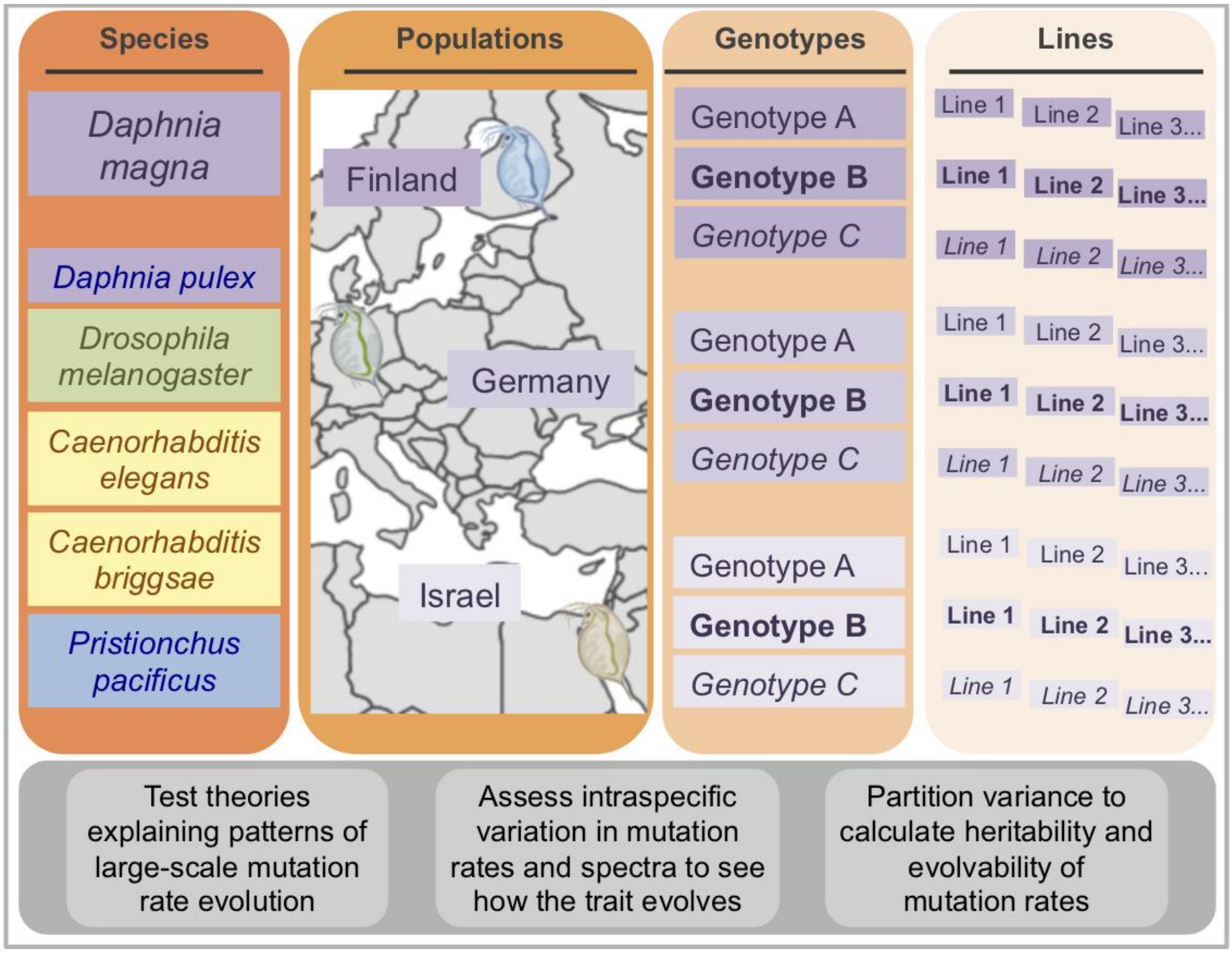

Figure S1. Measuring the means and variances of mutation rates at four levels, among species, populations, genotypes, and lines (represented by each panel) provides data for four levels of inference (bottom) about mutation rates, a critical parameter in evolutionary biology, and their evolution.

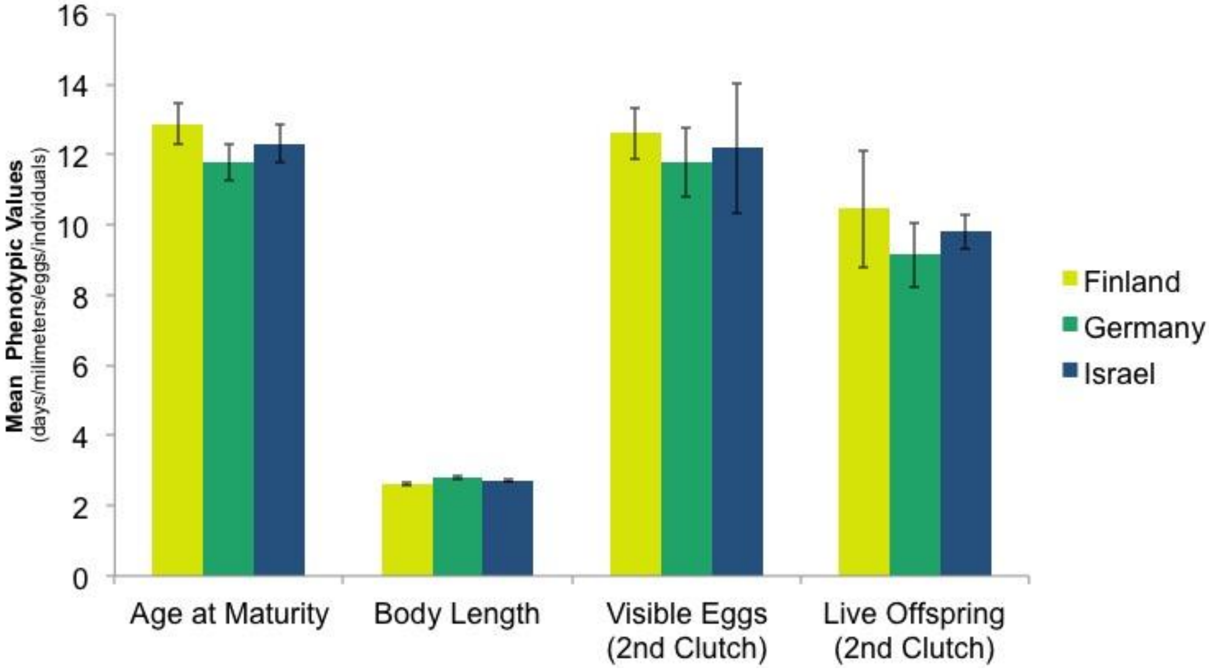

Figure S2. Mean phenotypic values from assays of life-history and fitness traits showing no major, consistent differences between ancestral genotypes (Finland [yellow], Germany [green] and Israel [blue]).

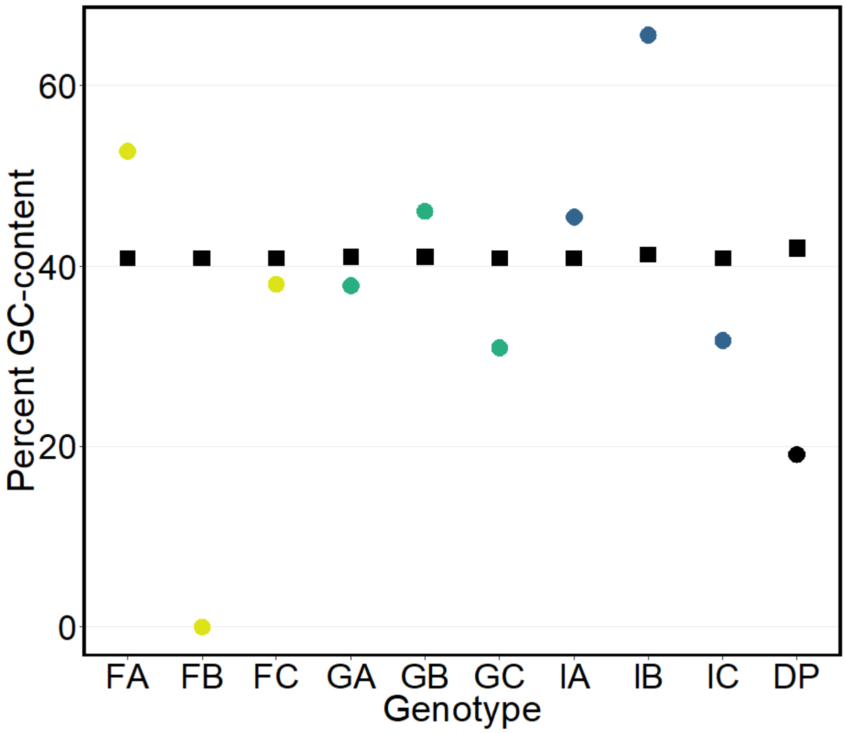

Figure S3. Observed (square) and expected (circle) equilibrium GC-content in each of the nine genotypes of *Daphnia magna* (Finland [yellow], Germany [green], and Israel [blue]). *Daphnia pulex* (DP) shown in black (from Flynn et al. 2016). Observed GC-content was calculated from the ancestral lines of each genotype. Expected equilibrium GC-content was calculated using the conditional rates of A/T -> C/G and C/G -> A/T substitutions.

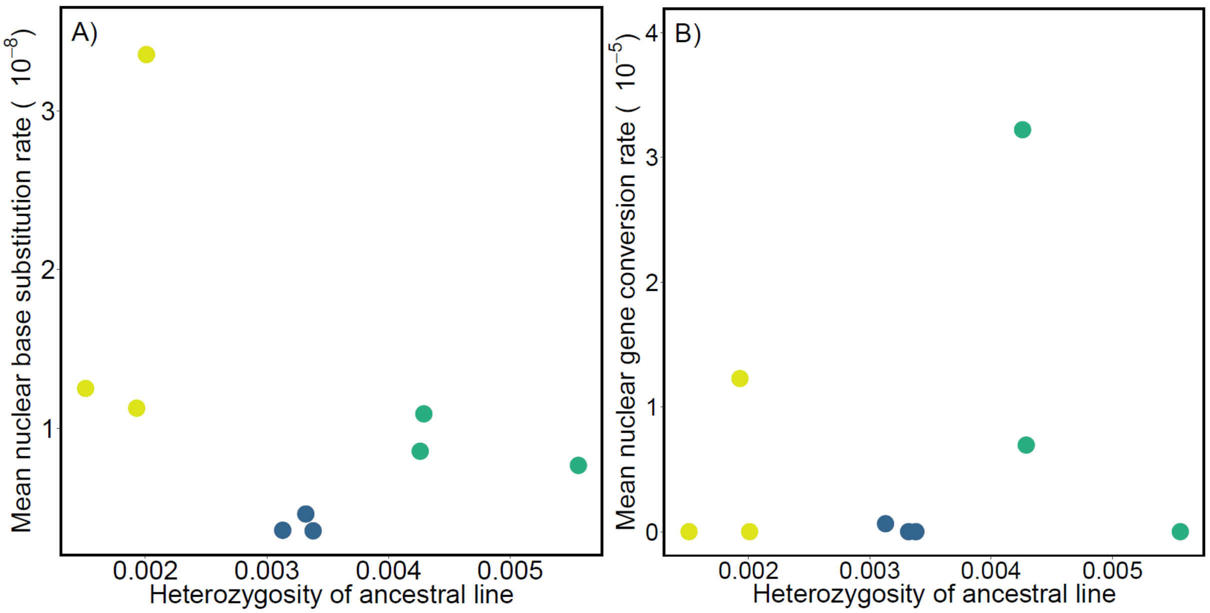

Figure S4. Heterozygosity of the ancestor for each genotype plotted against (A) nuclear base substitution mutation rate averaged across MA lines (B) nuclear gene conversion rate averaged across MA lines for each genotype of *D. magna* from Finland (yellow), Germany (green), and Israel (blue).

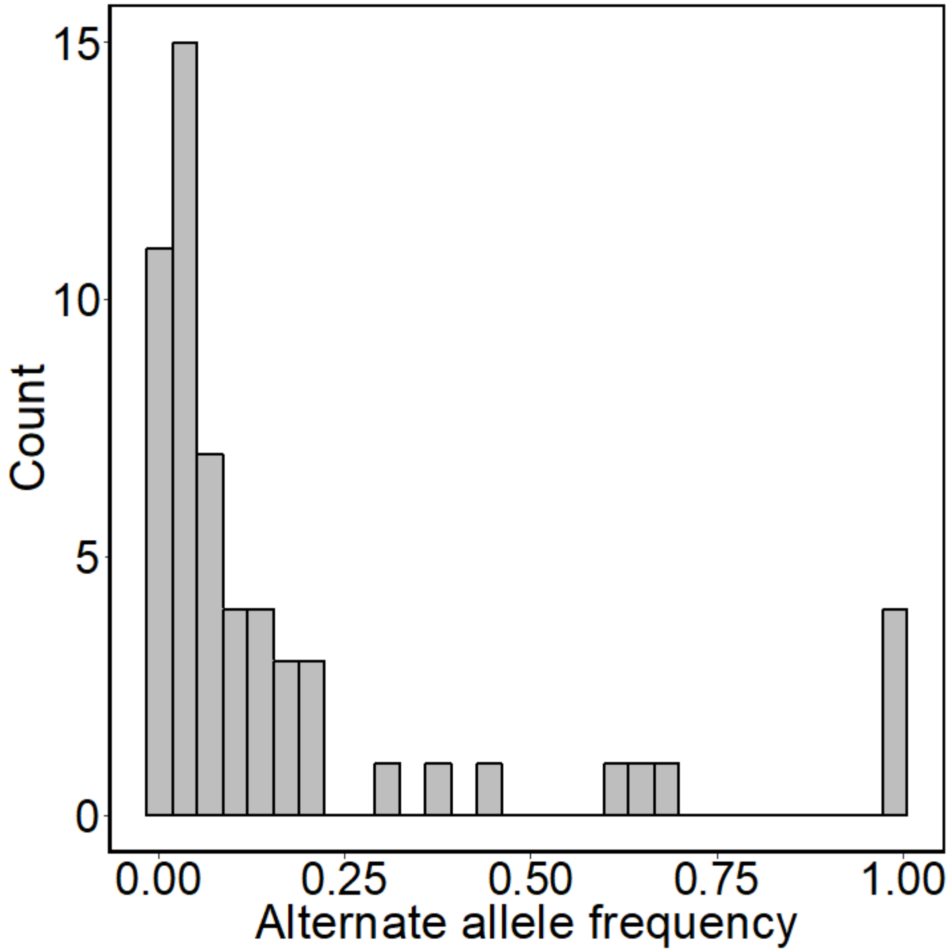

Figure S5. Histogram of the allele frequencies for all mitochondrial base substitutions observed across MA lines from for 9 genotypes of *D. magna*.

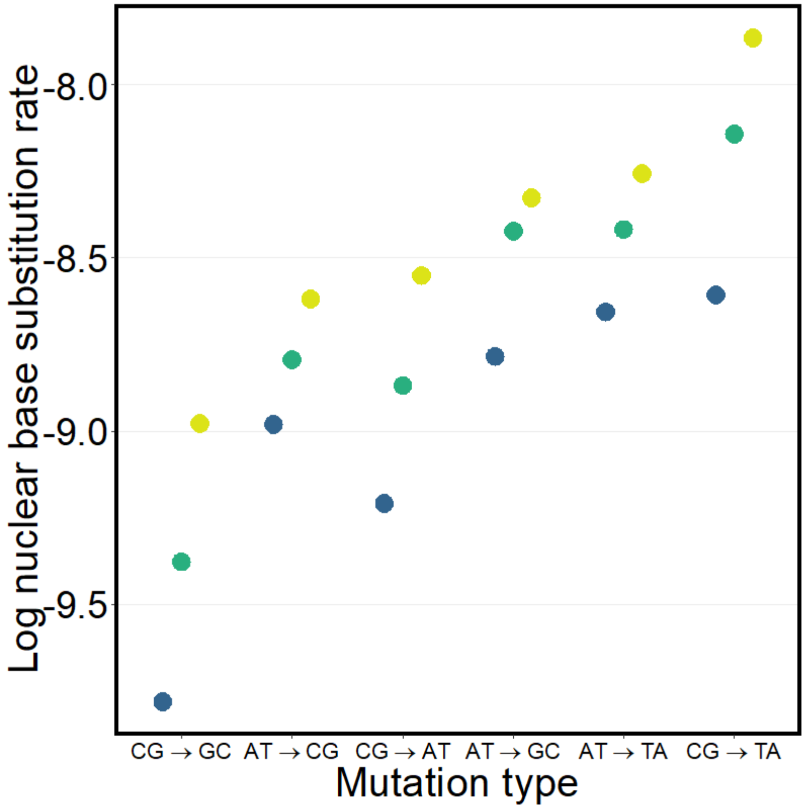

Figure S6. Conditional base substitution rates for each possible type of substitution averaged across MA lines for each population (Finland [yellow], Germany [green] and Israel [blue]).
