# Supplementary material for: High and Highly Variable Spontaneous Mutation Rates in Daphnia
Source: Mol Biol Evol. 2020 Jun 10;37(11):3258–66. doi: 10.1093/molbev/msaa142 (PMC7820357; doi:10.1093/molbev/msaa142)
Supplement: msaa142_Supplementary_Data [file msaa142_supplementary_data.zip › msaa142_supplementary_data/Supp_MM_Results_Figs_msaa142.pdf]

List of Supplementary Materials:

Supplemental Information: Methods and Materials

Supplementary Results

Supplementary Tables 1-12

Supplementary Figures 1-6

Supplementary References

Supplemental Information: Methods and Materials

Mutation Accumulation Lines

The nine *Daphnia magna* genotypes used in this experiment were initiated from three individuals collected from each of three populations (Germany, Finland, and Israel). Stock cultures for each genotype were maintained in 3 L jars containing 2 L of the medium AdaM (*34*) under a constant 16L:8D photoperiod, and fed *Scenedesmus obliquus* (a unicellular green algae) *ad libitum* (2-3 times per week). We used individual female descendants from each of the ancestral genotypes to establish mutation accumulation (MA) lines (*n* = 66 total) between October 2013 and April 2015. Individuals were maintained in 250 mL beakers containing 100 mL of ADaM, fed *Scenedesmus obliquus* at a concentration of 600,000 cells/mL, and maintained in a Percival environmental chamber under a 16L:8D photoperiod. Each line was propagated from generation to generation via single offspring descent by taking a single asexually-produced neonate from the second clutch and transferring it to a new beaker. Between transfers, media was changed weekly and feedings were performed to maintain algal cell concentration at approximately ~600,000 cells/ml. Backups were maintained in parallel with the focal lines in the event that the transferred individual died before reproduction or was a male. In the event that the focal line and all backups died before reproduction or were all males, the line was declared extinct and a new replicate line was established from the stock cultures. Immediate descendants of the individuals used to initiate the MA lines were both a) preserved for later sequencing (‘starting controls’ [SCs]) and used to initiate large population controls to run in parallel with the MA lines (‘extant controls’; (ECs]) to be sequenced at the end of the mutation accumulation period. Five clonal individuals from each line and control were flash frozen for DNA extractions.

DNA Extraction and Sequencing

For each sample, DNA was extracted (2 extractions per line) using the Zymo Quick-DNA Universal Solid Tissue Prep Kit (No. D4069) following the manufacturer’s protocol (DNA was also extracted from a few samples with the Qiagen DNeasy Blood and Tissue Kit, No. 69504). DNA quality was assessed by electrophoresis on 3% agarose gels and DNA concentration was determined by dsDNA HS Qubit Assay (Molecular Probes by Life Technologies, No. Q32851). The Center for Genome Research and Biocomputing at Oregon State University generated 94 Wafergen DNA 150 bp paired-end libraries using the Biosystems Apollo 324 NGS library prep system. Libraries were pooled based on qPCR concentrations across 16 lanes (2 runs) and sequenced on an Illumina Hiseq 3000. The average insert size was approximately 380bp.

Daphnia pulex sequence data

Additional sequence data was sourced from a recent MA experiment in *Daphnia pulex* (*7*) (Accession: PRJNA275628). The dataset consisted of paired-end short read Illumina libraries for MA lines originating from two isolates of *D. pulex*. Four MA lines from an obligate asexual genotype propagated by single-progeny descent for an average of 170 generations. Three MA lines from a cyclical parthenogenetic genotype from Slimy Log Pond, Oregon (referred to as “CYC”) which were propagated for an average of 85 generations.

Reads processing, de novo assembly

Reads from each sample were processed by trimming adaptor sequences (k=23, ktrim=r, mink=4, hdist=1, tpe, tbo), merging overlapping pairs (vstrict=t), and quality filtering (qtrim = rl, trimq=20, minlen=50) with BBTools version 37.36 (https://jgi.doe.gov/data-and-tools/bbtools/). All reads length <50 bp after trimming were discarded. To avoid alignment errors at the terminal ends of the mitochondrial reference (*D. magna*: Ebert, personal correspondence; *D. pulex*: accession AF117817 [*35*]), we produced a ‘rotated’ reference sequence, in which the first 5’ half of the sequence was moved to the 3’ end. This allowed reads which spanned the start-end junction to align correctly. In order to isolate the mitochondrial reads from the WGS libraries, reads were simultaneously aligned to the rotated and original mitochondrial reference as well as the nuclear reference (*D. magna*: accession LRGB00000000.1, *D. pulex*: accession ACJG00000000.1 (*36*)) using Bowtie2 (*37*) with the ‘very-sensitive’ preset. Reads in which both pairs aligned to the mitochondrial references were used for calling mitochondrial variants and the remaining reads for calling nuclear variants.

In order to map the nuclear reads, we first assembled genomes *de novo* for each of the nine starting *D. magna* genotypes (Table S11). First pass *de novo* assemblies were performed with SPAdes (*38*). To exclude mitochondrial contigs, we ran nBLAST (*39*) on the assembly against the *D. magna* mitochondrial reference and all contigs with significant hits were excluded. To remove major contaminants, we excluded assembled sequences with characteristics deviating substantially from the core set of contigs with high similarity to the *D. magna* reference genome (accession LRGB00000000.1), excluding contigs with average GC-content less than 30% or greater than 55%, or read coverage support <5x after mapping reads back to the preliminary assembly. To remove haplotigs putatively derived from the assembly of heterozygous regions into alternate alleles, we collapsed the filtered assemblies with *redundans* (*40*). Repetitive

regions of each assembly were identified and masked from downstream analysis using RepeatModeler (*41*) and RepeatMasker (*42*).

Mapping Variant Calling, and Validation

Nuclear genome

Nuclear reads for each sample were mapped to the genome assembly of their respective genotype using BOWTIE2 [*37*]; PCR and optical duplicates were removed with Picard MarkDuplicates (http://broadinstitute.github.io/picard). GATK *HaplotypeCaller* (*43*) was used to genotype each line separately. For each line, we required that all sites have a depth of coverage between 20 and 150. If the site was heterozygous, we additionally required: i) the site be biallelic, ii) the minor allele frequency be ≥0.2, iii) the second largest phred-likelihood score be ≥30, iv) at least one read of the alternate base be on the forward and backward strand. For each genotype, the number of callable sites (i.e. those where a new base substitution could be called) was defined as the number of sites where the ancestral lines and all MA lines passed the above requirements.

For each genotype, a base substitution mutation was called if one MA line had a new variant allele, while all other MA lines possessed the same genotype as the ancestral line. The new mutation was discarded if it was beside an indel and if the ancestral line or any of the unmutated MA lines possessed even a single read of the new variant allele when examined using Samtools *mpileup* (*44*). For each new substitution, we randomly sampled 10000 "truly" heterozygous sites in the genome (i.e., sites that are heterozygous in the starting controls and in all MA lines) with similar depth of coverage to the substitution and measured the proportion of reads supporting the alternate allele in this set. We compared the proportion of reads supporting the new substitution to this distribution (based on "true" heterozygous sites) and if the proportion fell between the 5th and 95th percentile of the proportions observed, the substitution was kept. Each new substitution was manually inspected with IGV (*45*) and we removed 128 substitutions where reads supported a substitution at the focal site, but showed polymorphism at one or more nearby sites. After these filters, we were able to recover 573 new substitutions. We subsampled 10 substitutions for verification with Sanger sequencing (see below and Table S12).

Mitochondrial genome

Mitochondrial reads were realigned to the original and rotated mitochondrial reference sequences using Bowtie2 (*37*). PCR and optical duplicates were removed with Picard and reads were locally realigned around indels using GATK *IndelRealigner* (*43*). Point mutations were called for each genotype separately and confined to the middle segment of the original and rotated alignments. At each site, the most common allele across all lines was considered to be the wildtype allele. The minor allele for each line was defined as the non-wildtype allele with the highest allele frequency. The minor allele frequency for MA line *i* was calculated as x_i / d_i, where x_i is the number reads containing the minor allele and d_i is the total number of reads. Mutant allele counts at each site were modeled as the outcome of a multinomial distributive process. If a site was truly unmutated, all non-wildtype reads are sequencing error and should be randomly distributed among MA lines in proportion to their depth of coverage. Thus, under this null hypothesis, we expect the non-wildtype reads for MA line i will have a multinomial probability $P_i = X * d_i / D$, where D and X are the total number of reads and non-wildtype reads summed across all MA lines, respectively. Variants were called by calculating the probability of observing a non-wildtype allele with allele frequency as extreme as that observed under the null hypothesis using a common approximation of the multinomial cumulative distribution function (github.com/fennerm/pmultinom; *46*). Q-values were calculated from the p-values across all genotypes using the qvalue package in R (*47*) and the false-discovery-rate (FDR) was set to 0.005.

To exclude pre-existing heteroplasmic sites as best we can, we excluded all sites in which p-values significantly differed from sequencing error in two or more MA lines using a binomial test. Sequencing error rates for each line was estimated from the nuclear alignments. Consensus sequences were produced for each genotype, and error rates were calculated as the total number of mismatches / number of assayed sites (*48*). To avoid confusing heterozygous sites with sequencing error, we ignored all positions with sequencing depth < 40 or minor allele frequency (MAF) > 0.2. However, it is impossible to distinguish between heterozygosity and sequencing error with complete certainty, so our estimates of sequencing error are likely to be slightly upwardly biased.

Phred-scaled strand bias was calculated for each site using Fisher's exact test following GATK (*43*) and variants with strand bias >60 were excluded. The high sequencing depth in our dataset allowed us to call mutations at extremely low frequencies (< 0.1 %). However, at such frequencies, it is difficult to distinguish germline mutations from somatic mutations and

sequencing error, so we required that mutations had a minimum allele frequency of 0.01. All mutations were visually inspected in IGV (*45*).

Base Substitution Rate Estimation

The nuclear base substitution mutation rate of each MA line was calculated as $\mu_{n,bs} = x_{bs} / (g * 2n)$ where x_{bs} represents the number of base substitution mutations that passed the above filters, g represents the number of MA generations and n number of callable sites (2*n* represents the number of diploid bases). The mitochondrial base substitution rate was calculated assuming neutrality (*33*) as $\mu_{m,bs} = \sum i\, f_i / (g\, n)$, where f_i is the allele frequency for mutation *i*, *g* is the number of MA generations, and *n* is the length of the mitochondrial genome.

Spectra Comparison

Each new base substitution mutation was categorized as one of either two types of transitions (CG→TA, AT→GC) or four types of transversions (CG→GC, CG→AT, AT→CG, AT→TA); treating strands symmetrically. The substitution rate for each category was conditional on its base composition in the reference genome. We calculated the rate of G/C → A/T (*v*) and the rate of A/T → G/C (*u*) to calculate the expected GC-content of the genome at equilibrium as $u / (u+v)$. To examine the effect of local context on mutation rates, we examined the substitution rate of the central nucleotide at all 32 possible 3-bp contexts, conditional on base composition (i.e. 16 contexts each for cases where the central nucleotide was C (or G) and A (or T)).

Nuclear Gene Conversion Rate Estimation

For each genotype, a heterozygous site that exhibited a loss of heterozygosity (LOH) event was called if the ancestral line and all but one MA line were heterozygous for the same genotype, while the focal MA line was homozygous for one of the pre-existing alleles. The site was discarded if the homozygous site contained even one read of an alternate allele when examined using Samtools *mpileup* (*44*). To increase our confidence that a LOH event was due to gene conversion rather than a hemizygous deletion, we required the depth of coverage to be similar to other sites in the genome and to the same site in lines that did not undergo LOH. For each line we standardized the depth of coverage of sites by dividing by the average coverage across all callable sites. Our initial filters to detect LOH sites in MA lines required that the standardized coverage at the LOH site (D_{loh}) be at least 0.70 and the ratio of D_{loh} to the average standardized coverage in the SC and the MA lines that remained heterozygous (D_{het}) be at least 0.8. LOH sites were also discarded if they did not lie within a gene conversion tract, which we define as a

region containing at least two LOH sites uninterrupted by a heterozygous site. From this first round of filtering, we found 652 LOH sites. We chose 9 of these LOH sites for validation and were able to validate 5 through Sanger sequencing (see below and Table S12).

Thus, based on the validated sites, we modified our filter to require D_{loh} be between 0.75 and 1.45, the ratio D_{loh} / D_{het} be between 0.8 and 1.3, and LOH sites reside within a tract with at least two LOH sites uninterrupted by heterozygous sites. We define the minimum size of the tracts as the distance between the most upstream and most downstream gene conversion sites and the maximum size of the tracts as the distance between the two nearest heterozygous sites that flanked the tract. Each gene conversion site was manually inspected in IGV[39] and discarded sites were not used to calculate mutation rates in any category. The gene conversion rate for each MA line was calculated as $\mu_{n,g} = x_g / (g * n)$, where x_g represents the number of gene conversion sites, g represents the number of MA generations and *n* represents the number of heterozygous sites in the ancestral genotype. After this set second of filters, we found 307 sites that experienced gene conversions (including the five validated by Sanger sequencing; Table S12) within 123 gene conversion tracts spread among 39 of the 66 MA lines (Table S8A).

However, we noticed that coverage around these gene conversion sites were quite variable so we applied a final set of filters based on the depth of coverage and the length of each tract. We required that the minimum gene conversion tract (i.e., the region between the most upstream and downstream gene conversion site of the tract) contained no more than 10% of sites with less than 10x coverage. This filter eliminated 23 gene conversion tracts, including those that contained four of the five Sanger sequence validated gene conversion sites. The one remaining validated site occurred within a tract with minimum length of 32 bp. Based on this, we applied an additional filter requiring that the minimum track length be at least 32 bp. After this final set of filters, we obtained 35 gene conversion sites within 13 gene conversion tracts among six of the 66 MA lines (Table S8A).

Base substitution and loss of heterozygosity site validation

Ten of the 573 nuclear base substitution mutations identified in the short-read data after all filters as well as nine of the 652 LOH mutations identified after the first round of filters were validated using PCR, cloning, and Sanger sequencing to accurately determine the sequence of between 8 and 16 colonies per mutation. PCR was performed in 25 µl reactions containing Qiagen Taq PCR Master Mix; 500 nM forward and reverse primers (individually designed using

Primer3 for ~300 bp amplicon surrounding the desired mutation site); and 2 µl of DNA from the respective population and MA line. The samples were then loaded into the BioRad T100 Thermal Cycler and underwent following program: 1) 94 °C for 3 min, 2) 94 °C for 30s, 3) 50 °C for 30s, 4) 72 °C for 1 min, 5) repeat from step 2 twenty-four more times, 6) 72 °C for 10 min, and 7) 4 °C hold indefinitely. The PCR products were mixed with 1X EZ-VISION Three DNA Dye Loading Buffer (Thomas Scientific) and were run on a 1% agarose gel alongside NEB 100 bp ladder using gel electrophoresis.

Cloning reactions consisted of 4 µl PCR product, 1 µl TOPO TA Cloning Salt Solution (Invitrogen), and 1 µl TOPO TA Cloning Vector (Invitrogen). This reaction sat at room temperature for 5 min before being put on ice either temporarily or overnight. Next, 2 µl of the cloning reaction was added to a thawed vial of Invitrogen OneShot TOP10 Chemically Competent E. coli; the solution was gently mixed, incubated on ice for 5-30 min, incubated at 42 °C for 30 sec, returned to ice, mixed with 250 µl SOC media, and incubated in a Benchmark Incu-Shaker Mini at 37 °C and 200 rpm for one hour. Then each solution was plated twice on 50 µg/ml Kanamycin LB plates using 50 µl and 75 µl of the solution and colonies were allowed to grow overnight at 37 °C in a Digital Laboratory General Purpose Incubator (SHELL LAB). Eight to sixteen transformed (visually white) colonies per mutation were picked and suspended in 25 µl TE (Tris-EDTA Buffer). These samples were then boiled at 95 °C for 5 min, vortexed, cooled, and centrifuged for 1 min. The supernatant was isolated and utilized as the DNA for a second round of PCR using M13 primers, performed as above with an annealing temperature of 45 °C.

Sequencing reactions were performed using BigDye Terminator v3.1 (Applied Biosciences), BigDye Terminator v1.1 v3.1 1X Sequencing Buffer, 200 nM M13 forward primer (TGTAAAACGACGGCCAGT), and 4 µl of PCR product from a single colony (10 µl total) using the following program: 1) 96 °C for 2 min, 2) 96 °C for 30s, 3) 47 °C for 30s, 4) 60 °C for 4 min, 5) repeat from step two 24 more times, and 6) 4 °C hold indefinitely. Reactions were shipped to ACGT Inc. for visualization. Chromatograms were analyzed using MEGA7 (*49*) to align to reference genomes for each MA line and locate mutations.

Pairwise-genetic distance within populations

We mapped reads from each of the ancestral lines to a common *D. magna* reference (Ebert: personal correspondence) using BWA [50]. We then used GATK *HaplotypeCaller* (*43*) to assign genotypes for each site of each line separately. For each line, we required that all sites have a

depth of coverage between 20 and 150. If the site was heterozygous, we additionally required i) the site be biallelic, ii) the minor allele frequency be ≥0.2, iii) the second largest phred-likelihood score be ≥30, and iv) at least one read possessing the alternate base on the forward and backward strand. We then applied one more filter for each population separately (Finland, Germany, Israel), requiring that a site be genotyped at all three ancestral lines from a given population. We were able to call 95198658, 81635224 and 74825921 sites for ancestral lines from Finland, Germany and Israel, respectively. We then calculated pairwise genetic distances between each of the three ancestral lines within each population. Pairwise-genetic distance was calculated by summing the number of sites between two samples that differed in genotype and dividing by the number of called sites (Table S7). Pairs of sites that are homozygous for different alleles (homozygous differences) are weighted twice as much (1) as pair of sites where one sample is homozygous and the other sample is heterozygous (0.5; heterozygous differences).

Nuclear microsatellite mutation rates

Microsatellite mutation rates were obtained from a previous study on the same *D. magna* MA lines, but restricted to only 6 of the genotypes FA, FC, GA, FC, IA, IC (*11*). Briefly, we used the software k-Seek (*51*) to scan for microsatellite content in the sequence reads of each ancestor and MA line. k-Seek detects tandemly repeating motifs(k-mers) with lengths 1-20 bp spanning ≥50 bp on a given read, allowing for one base pair mismatch per repeat unit. To obtain more reliable mutation rates, we required that k-mer have at least six copies in the ancestral genotype and at least two copies in each of the descendent MA lines. Depending on the genotype being examined, there were 60 to 79 k-mers that fulfilled this requirement. Per copy mutation rates for each k-mer type *i* of MA line *j* was calculated as $u_{i,j} = (c_{i,j} - c_{SC,j}) / (c_{SC,j} * g_i)$, where $c_{i,j}$ and $c_{SC,j}$ represents the copy number of k-mer *j* at MA line *i* and the ancestral line, respectively, and g_i represents the number of MA generations. Since k-mer copy numbers can decrease or increase from mutation, we took the absolute value of $u_{i,j}$ to represent the propensity for mutation. Then for each MA line, we calculated the absolute microsatellite mutation rate ($|\mu_{n,ms}|$) by averaging $|u_{i,j}|$ across all k-mers.

Statistical Analyses

All analyses were performed using R (*47*). To determine if nuclear base substitution rates differed between populations, we fitted a linear mixed effects models on the log-transformed rates with population, and genotype (nested within population) as the fixed and random effects,

respectively; allowing for different variances between genotypes. If population had a significant effect, we performed post-hoc Tukey tests to determine the significance of pairwise comparisons. The variance in mutation rates were partitioned to the components that were among (V_a) and within (V_e) genotypes. Mutational heritability (H_2) was estimated as the ratio of the per generation change in the among genotype variance ($V_m = V_a/2g$, where g is the average number of MA generations) to the within genotype variance (V_e) (*52*). Evolvability was estimated as V_m/x^2, where x^2 is the square of the mean mutation rate. 95% confidence intervals for heritability and evolvability were estimated by bootstrapping 1000 times. We used Kruskal-Wallis test to determine if mitochondrial base substitution rates differed between populations. Chi-square tests were used to determine whether the spectra of substitutions differed between populations. To analyze the effect of local context on the conditional base substitution rates, we averaged the rates across MA lines of each genotype for each of the 32 possible 3-bp context and excluded FB because it had too few mutations. We then fit a linear mixed effects model on the conditional rates with the initial state of the central nucleotide (A or C), the 16 flanking nucleotide context and population as fixed effects and genotype (nested within population) as a random effect, allowing for different variances between genotypes. To examine the relationship between different types of mutations we calculated pairwise Pearson correlations across MA lines; 95% confidence intervals were obtained by bootstrapping.

Supplementary Results

We generated and aligned short read datasets of whole genome sequence (WGS) generated for MA lines (*n* = 66) from nine ancestral *D. magna* genotypes, three each from Finland, Israel and Germany to an average depth of 50x (nucDNA) and 4932x (mtDNA). In addition, short read datasets from *D. pulex* (NCBI BioProject Accession: PRJNA275628 [7]) were aligned to the mitochondrial reference (Accession: NC_000844) to an average depth of 1608x. Mapped reads were used to call mutations and calculate base substitution mutation rates in the nuclear and mitochondrial genome, the spectra of mutations, and gene conversion rates in the nuclear genome. On average, the MA lines underwent 12 generations of mutation accumulation (Table S1B).

Nuclear base substitution rates

We detected a total of 573 spontaneous nuclear base substitutions in the *D. magna* MA lines. There were 11 sets of multinucleotide mutations (substitutions occurring within 50 bp of each other) occurring within FB, FC, GA, GC, IA and IC MA lines. Nine of the eleven multinucleotide mutations were pairs of occurred within 5 bp of each other. One pair of substitutions in GC6 occurred within 22 bp of each other and one triplet in IC10 spanned 41 bp. Wet bench validation of 10 of the mutations (using PCR, cloning, and Sanger sequencing) verified 10 out of 10 base substitutions identified using our bioinformatic pipeline. Based on the observed events, the mean base substitution mutation rate ($\mu_{n,bs}$) is 8.96 x 10^{-9}/bp/generation (bootstrap 95% CI: 6.66-11.97 x 10^{-9}/bp/generation) across MA lines (Table S1A). We did not detect any base substitutions in MA line IB5 (Table S1B). Ignoring IB5, the MA lines possessing the highest and lowest rates were FB11 (7.83 x 10^{-8}/bp/generation) and GB4 (1.16 x 10^{-9}/bp/generation), which spanned over an order of magnitude. The rate for FB11 was more than twice as high as the next highest line-specific rate across the entire experiment (Table S1B), which might indicate a mutation that increased mutation rates (a “mutator allele”) occurred in this line during the mutation-accumulation period. As one would expect, the number of generations of mutation accumulation that could be completed (before extinction) was lower in lines that, ultimately, exhibited the highest mutation rates, although we do not thing this presents an additional bias to our estimates given that new lines derived from those ‘high mutation rate’ genotypes were then initiated again to try and keep the sample sizes as even as possible. To be complete, we also calculated genome-wide estimates of the mutation rate and mutation rates based on absolute time (per bp per day; Table 1).

The mean $\mu_{n,bs}$ was estimated for each of the nine genotypes by averaging across MA lines (ranging from 3.35 x 10^{-8} /bp/generation in FB and 3.57 x 10^{-9}/bp/generation in IB; Table S1A). Using a mixed effects linear model with population as a fixed effect and genotype as a random effect that can possess different variances, we found that populations vary significantly in their mutation rates ($F_{2,6}$ = 20.36, p = 0.0021). Genotypes from Finland tend to have higher substitution rates (1.54 x 10^{-8}/bp/generation) than those from Germany (9.06 x 10^{-9}/bp/generation) which tend to have higher rates than those from Israel (3.93 x 10^{-9}/bp/generation). Post-hoc Tukey tests revealed that $\mu_{n,bs}$ for genotypes from Finland and Germany did not differ, but were significantly higher than rates for Israel (Figure 1B).

We observed that $\mu_{n,bs}$ for genotypes within Finland varied more than genotypes within Germany and genotypes within Israel (coefficient of variation in $\mu_{n,bs}$: 0.654, 0.185, 0.153 for Finland, Germany, and Israel, respectively). To test if variation in mutation rates among genotypes within a population simply reflects patterns of genetic-relatedness among genotypes, we looked for correlations between the variance in mutation rates and the pairwise-genetic distance between genotypes. We observed no relationship, which suggests the intraspecific variation we observe is not simply due to relatedness (Table S7).

Nuclear gene conversion rates

Initially, we found 307 gene conversions (including the five validated by Sanger sequencing) within 123 gene conversion tracts spread among 39 of the 66 MA lines (Table S8A). The nuclear gene conversion rate ($\mu_{n,g}$) averaged across all *D. magna* MA lines for this sets of sites was 5.17 x 10^{-6}/heterozygous site/generation, which is comparable but an order of magnitude lower than the rate estimated in *D. pulex* (*7*, *24* [the rate before their final filter]). Thus, we applied a more stringent set of filters that required minimum gene conversion tracts be at least 32 bp long and contain no more than 10% of sites with less than 10x coverage. Ultimately, we found evidence for 35 gene conversion sites within 13 gene conversion tracts among six of the 66 MA lines (Table S8A, S8B), resulting in a mean rate of gene conversion ($\mu_{n,g}$) across all *D. magna* MA lines of 6.13 x 10^{-7}/heterozygous site/generation. This rate is two orders of magnitude lower than the *D. pulex* rate estimated in Keith et al. (2016) but one order of magnitude greater than the rate estimated in Flynn et al. (2016). For the MA lines where we detected gene conversions, $\mu_{n,g}$ range from 5.09 x 10^{-7}/heterozygous site/generation in IA2 to 1.72 x 10^{-5}/heterozygous/site/generation in GB8. We also estimated gene conversion rates for

control lines following the same procedure as used for the MA lines. Two control lines for each of the 9 ancestral genotypes (n = 18) were maintained in large populations (hundreds to thousands of individuals) for an average of 50 generations before samples were sequenced. We were able to detect 7 gene conversion sites and 3 gene conversion tracts in 3 of the 18 control lines, which resulted in average gene conversion rate of 3.43 x 10^{-8}/ heterozygous /site /generation. This order of magnitude reduction in gene conversion rate compared to MA lines suggests gene conversion events are relatively deleterious and are purged when natural selection can act in larger populations. Heterozygous sites that experienced gene conversion events were all tightly clustered reflected by the short minimum lengths in the 13 gene conversion tracts of MA lines (32 to 98 bp) and the 3 tracts in EC lines (49 to 56 bp). The majority of the regions contained within the maximum tract (i.e., between the closest upstream and downstream heterozygous site) possessed greater than 10% of sites with coverage lower than 10x (Table S8B).

Mitochondrial base substitution rate

A total of 54 mitochondrial base substitutions were identified across all *D. magna* MA lines, with no substitutions detected in 32 of the 66 lines. As expected, most new mutations were at low allele frequencies (Figure S5). Averaging across all MA lines, the spontaneous mitochondrial base substitution rate ($\mu_{m,bs}$) is 8.7 x 10^{-7}/bp/generation (bootstrap 95% CI: 4.40-15.12 x 10^{-7}/bp/generation), which is an order of magnitude higher than the previous highest reported rate of 9.7 x 10^{-8}/bp/generation from *Caenorhabditis elegans* (*3*, *8*). The high sequencing depth in our data gave us the statistical power to call mutations at extremely low frequencies (<0.1 %). However, at such frequencies it is difficult to distinguish germline mutations from somatic mutations and sequencing error, so we required that mutations have a minimum allele frequency of 0.01. Even with this restriction, our ability to call variants accurately is likely to be lower as allele frequencies decrease. If we were to conservatively exclude all mutations below 0.1 allele frequency, our estimated substitution rate becomes 6.64 x 10^{-7}/bp/generation (bootstrap 95% CI: 2.50-12.85 x 10^{-7}/bp/generation). Excluding the MA lines without new base substitutions, $\mu_{m,bs}$ ranged from 1.74 x 10^{-5}/bp/generation in GC8 to 3.94 x 10^{-8}/bp/generation in IC1, spanning three orders of magnitude. MA lines from Germany (mean = 1.53 x 10^{-6}/bp/generation) tend to have higher substitution rates than those from Finland (mean = 5.86 x 10^{-7}/bp/generation) and Israel (mean = 4.21 x 10^{-7}/bp/generation) although, unlike the nuclear

base substitution rates, we did not find a significant difference in mutation rates across populations (χ^2 = 1.2, df = 2, p < 0.001).

It is worth noting, while MA experiments allow for the complete bottleneck of the nuclear genome via single-progeny descent, the mtDNA cannot be completely bottlenecked because there are many copies of the genome scattered throughout each cell's mitochondrial matrix (*53*). While mean mtDNA copy number per cell can be estimated empirically based on depth of sequencing at mtDNA loci (normalized by depth of sequence at single copy nuclear genes), the size of the genetic bottleneck during gametogenesis and the effective number of mtDNA ($N_{e,\ mt}$) cannot be estimated in this way. Differences in the size of the bottleneck, $N_{e,\ mt}$, or the distribution of fitness effects for mtDNA mutations among genotypes or populations could mask or enhance differences in the actual mutation rate (mistakes due to polymerase faults or unrepaired DNA damage) thus making mtDNA mutation rate estimates more challenging, even in a mutation-accumulation experiment.

Comparison to base substitution rates in D. pulex

Estimates of $\mu_{n,bs}$ for *D. pulex* are 2.3 x 10^{-9}/bp/generation (*24*), 4.33 x 10^{-9}/bp/generation (cyclical parthenogenetic genotyp [7]), 1.02 x 10^{-8}/bp/generation (asexual genotype [7]) and 1.55 x 10^{-9}/bp/generation (*54*). These estimates span an order of magnitude and possess a mean of 4.59 x 10^{-9}/bp/generation and a standard deviation of 3.91 x 10^{-9}/bp/generation. Estimates of $\mu_{n,bs}$ for the nine genotypes of *D. magna* also span an order of magnitude (3.57 x 10^{-9} to 3.55 x 10^{-8}/bp/generation) but has higher mean (8.96 x 10^{-9}/bp/generation) and standard deviation (9.19 x 10^{-9}/bp/generation).

Using the *D. pulex* sequence data from the MA lines of (*7*), we found three mitochondrial base substitutions and estimated $\mu_{m,bs}$ to be 8.54 x 10^{-8}/bp/generation which was higher than the previous *D. pulex* estimates of 2.0 x 10^{-8}/bp/generation (sexual genotype [*55*]) and 4.3 x 10^{-8}/bp/generation (asexual genotype [*55*]). All but one of our *D. magna* genotypes possessed a higher mitochondrial base substitution mutation rate than the *D. pulex* mean (Figure 2; Table 1). The sequencing coverage was considerably higher in our *D. magna* data than the *D. pulex* data (*7*). To check that the observed difference in mutation rates was not the result of different coverage, we down-sampled both datasets to an arbitrary depth of 500x and repeated our mutation calling procedure. The base substitution mutation rate in *D. magna* remained larger

than in *D. pulex* (bootstrapped $p < 0.001$; 10,000 replicates), indicating that differences in sequencing depth alone do not explain the mutation rate differences.

Spectra and local context of base substitution mutations

For each genotype, we counted the occurrence of the six possible types of base substitution mutations, treating strands symmetrically. Summing the counts across all genotypes, we found that the six possible types of substitutions vary in frequency ($\chi^2 = 337.7$, df = 5, $p < 0.001$). The majority of substitutions were CG->TA transitions (Table S3A) and the transition to transversion ratio in the nuclear genome is 1.54, which is similar to previous findings (*3*). We did not find a significant difference in the substitution spectrum between populations ($\chi^2 = 8.8$, df = 10, $p = 0.5514$), unless local context was taken into account.

To examine the effect of local context on mutation rates, we calculated the substitution rate of the central nucleotide at all 32 possible 3-bp contexts, treating strands symmetrically. We excluded genotype FB from our statistical analyses because it had few mutations (22 in total) and would give a poor representation for the distribution of mutation rates across the 32 contexts. We performed a mixed effect linear model with the central nucleotide (A or C), the 16 types of local contexts (flanking nucleotides), and population as fixed effects and genotype as a random effect. We found when the center nucleotide was initially C (or G), the mutation rate was significantly higher than when the center nucleotide was A (or T) ($F_{1,232} = 25.6$, $p < 0.0001$; Table S5). We also found that the local context had a significant effect on mutations rates ($F_{15,232} = 3.6$, $p < 0.0001$). To examine if CpG sites affected mutation rates, we narrowed our analysis to the contexts where the center nucleotide was C (or G) and calculated the mean rates for contexts that contained a CpG site versus those that did not for each genotype (Table S5). A paired t-test showed that CpG sites have a significantly lower mutation rate than non-CpG sites ($t_7 = 5.29$, $p = 0.0011$).

In the mitochondria, summing the counts across all genotypes for each of the six possible types of substitutions, we found that the six possible types were also significantly non-randomly distributed ($\chi^2 = 44.9$, df = 5, $p < 0.001$). A transition to transversion ratio of 2.86 shows a stronger bias for transitions, with a similar number of CG->TA (22) and AT->GC (18) transitions. This Ts:Tv is in the middle of a wide range observed across species (Table S3C). After controlling for reference base composition, the ratio of CG->TA to AT->GC substitutions was 2.49:1, showing a bias towards the former.

Observed GC-content in the nuclear genome was very similar in of each of the sequenced ancestors (~ 41%; Table S6). We used the substitution rate for C/G->A/T and A/T->C/G (conditional on base composition; Figure S6) to estimate the equilibrium GC-content for each genotype and found a wide range (31-66%; Table S6). Flynn et al. (2016) showed that the expected equilibrium GC-content in *D. pulex* (19.1%) was much lower than observed (42%), similar to reports for several algae (*56*). Given that the range of expected equilibrium GC-content was much larger than the observed range, evolutionary forces other than mutation must be acting to shape the GC-content in the nuclear genome in this species. Using the conditional substitution rate for C/G->A/T and A/T->C/G, we estimate the equilibrium GC-content in the mitochondria to be 40.6% which was slightly higher than the observed GC-content of 32.9% in the reference genome.

Large-scale patterns in rate variation

To see if there was any evidence in support of mutation rates varying as a function of either a) age-at-maturity (*18*) or b) genetic "quality" (*15*), we plotted phenotypic data for typical fitness traits (Figure S2). There are no differences among the genotypes originating from Finland, Germany, and Israel either for age at maturity or any of three common fitness traits (body size, egg number, and number of offspring (Figure S2).

We tested for correlations among the rate estimates to see if they co-vary positively or negatively. We observe no correlation (ρ = -0.02, t_{64} = -0.18, p = 0.85) between the mitochondrial and nuclear substitution rates (Table S10). We also looked at correlations between base substitution mutation rates and microsatellite mutation rates ($\mu_{n,ms}$) for a subset of the same *D. magna* MA lines analyzed in a previous study (*11*). Since microsatellite copy numbers can increase or decrease due to mutation, we used the absolute value of the mutation rates as a measure for the propensity for mutation ($|\mu_{n,ms}|$). Each value of $|\mu_{n,ms}|$ represents the absolute mutation rate averaged across 60 to 79 types of microsatellites, depending on the genotype examined ([11] for details). We observed a moderate and significant positive correlations between $\mu_{n,bs}$ and $|\mu_{n,ms}|$ (ρ = 0.61, t_{45} = 5.1, p < 0.0001) but a weak non-significant correlation between $\mu_{m,bs}$ and $|\mu_{n,ms}|$ (ρ = 0.11, t_{45} = 0.71, p = 0.48). When we calculated the correlation between $|\mu_{n,ms}|$ and $\mu_{n,bs}$ for each population individually, we found that the correlation was non-significant for Finland lines (ρ = 0.48, t_{13} = 1.96, p = 0.07), but significantly positive for Germany (ρ = 0.67, t_{14} = 3.33, p = 0.005) and Israel lines (ρ = 0.73, t_{14} = 3.99, p = 0.001).

Supplementary Tables and Figures

Supplementary Tables

Supplementary Tables S1 through S12 are all in a workbook, uploaded separately as a pdf and available at:
https://www.dropbox.com/s/oau0bwnk6aqortx/SuppTables092319.xls?dl=0

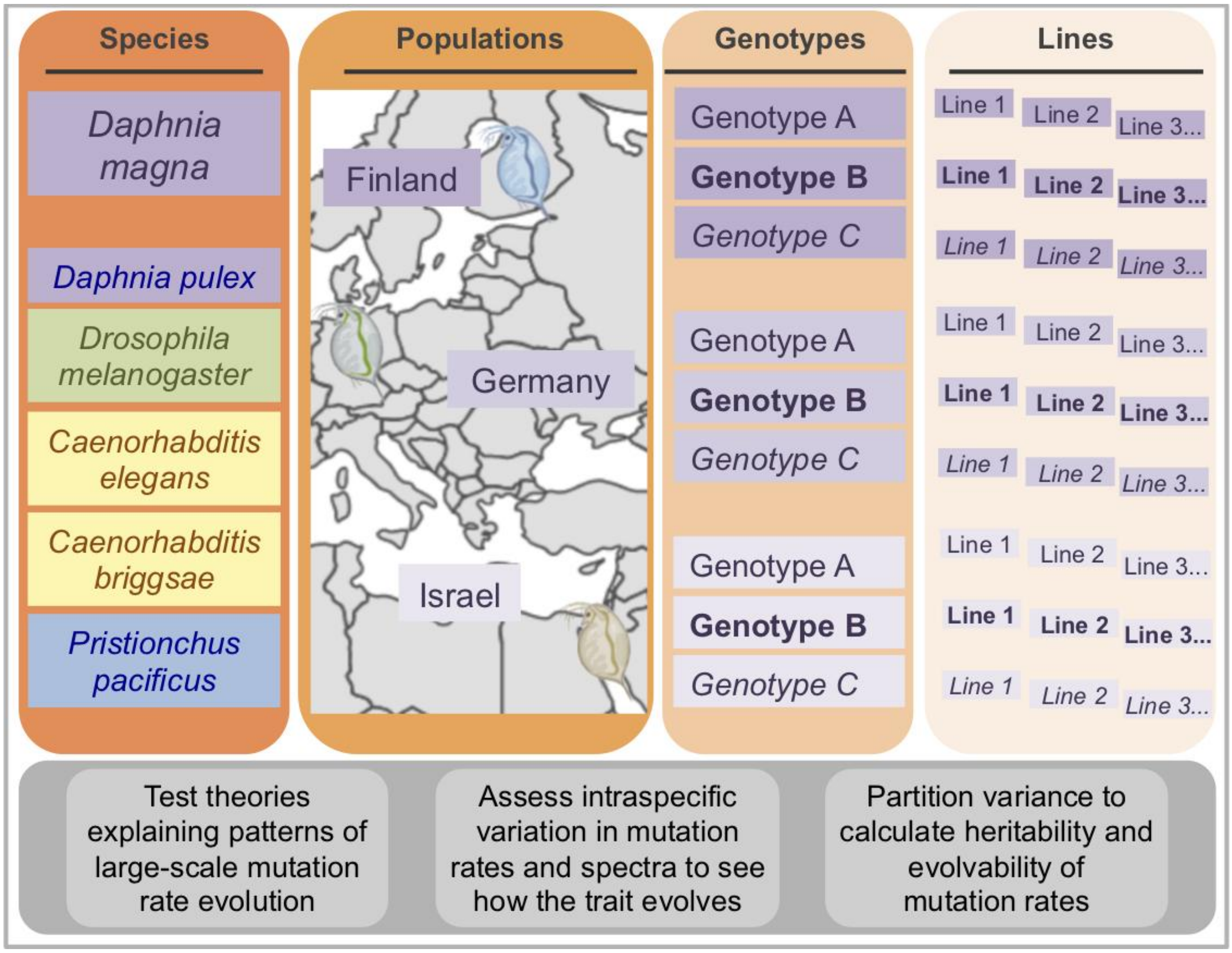

Figure S1. Measuring the means and variances of mutation rates at four levels, among species, populations, genotypes, and lines (represented by each panel) provides data for four levels of inference (bottom) about mutation rates, a critical parameter in evolutionary biology, and their evolution.

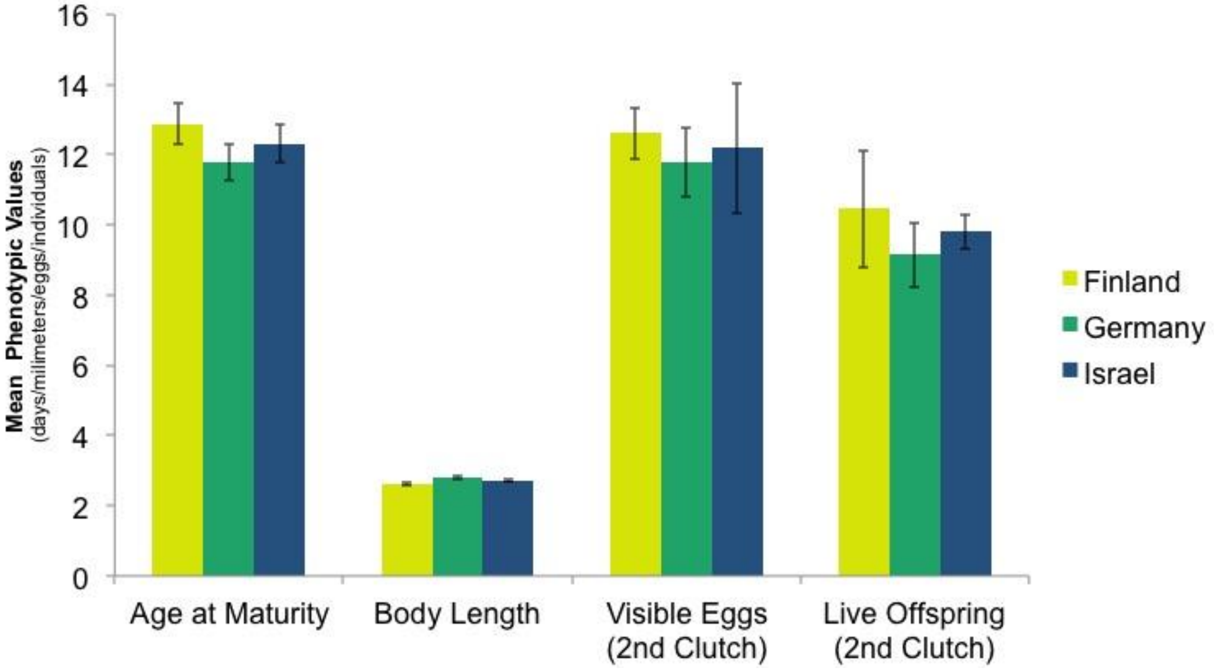

Figure S2. Mean phenotypic values from assays of life-history and fitness traits showing no major, consistent differences between ancestral genotypes (Finland [yellow], Germany [green] and Israel [blue]).

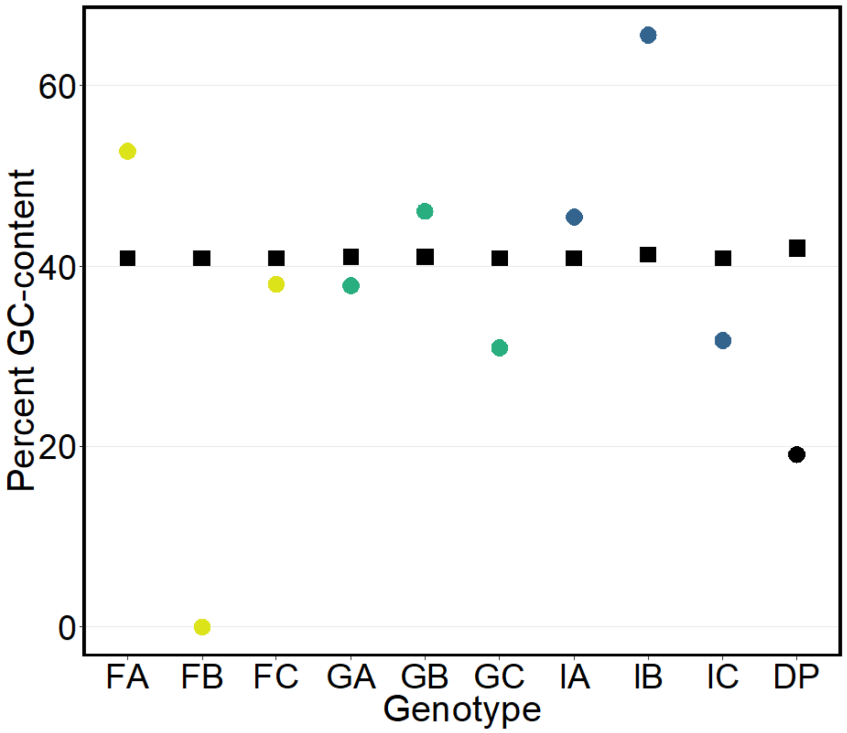

Figure S3. Observed (square) and expected (circle) equilibrium GC-content in each of the nine genotypes of *Daphnia magna* (Finland [yellow], Germany [green], and Israel [blue]). *Daphnia pulex* (DP) shown in black (from Flynn et al. 2016). Observed GC-content was calculated from the ancestral lines of each genotype. Expected equilibrium GC-content was calculated using the conditional rates of A/T -> C/G and C/G -> A/T substitutions.

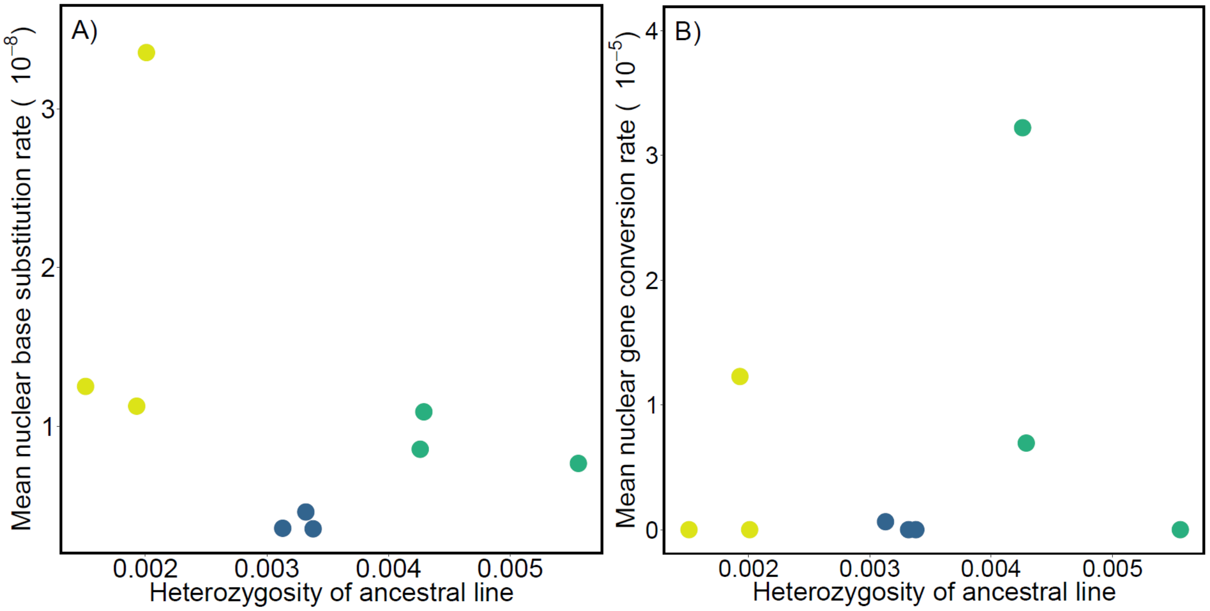

Figure S4. Heterozygosity of the ancestor for each genotype plotted against (A) nuclear base substitution mutation rate averaged across MA lines (B) nuclear gene conversion rate averaged across MA lines for each genotype of *D. magna* from Finland (yellow), Germany (green), and Israel (blue).

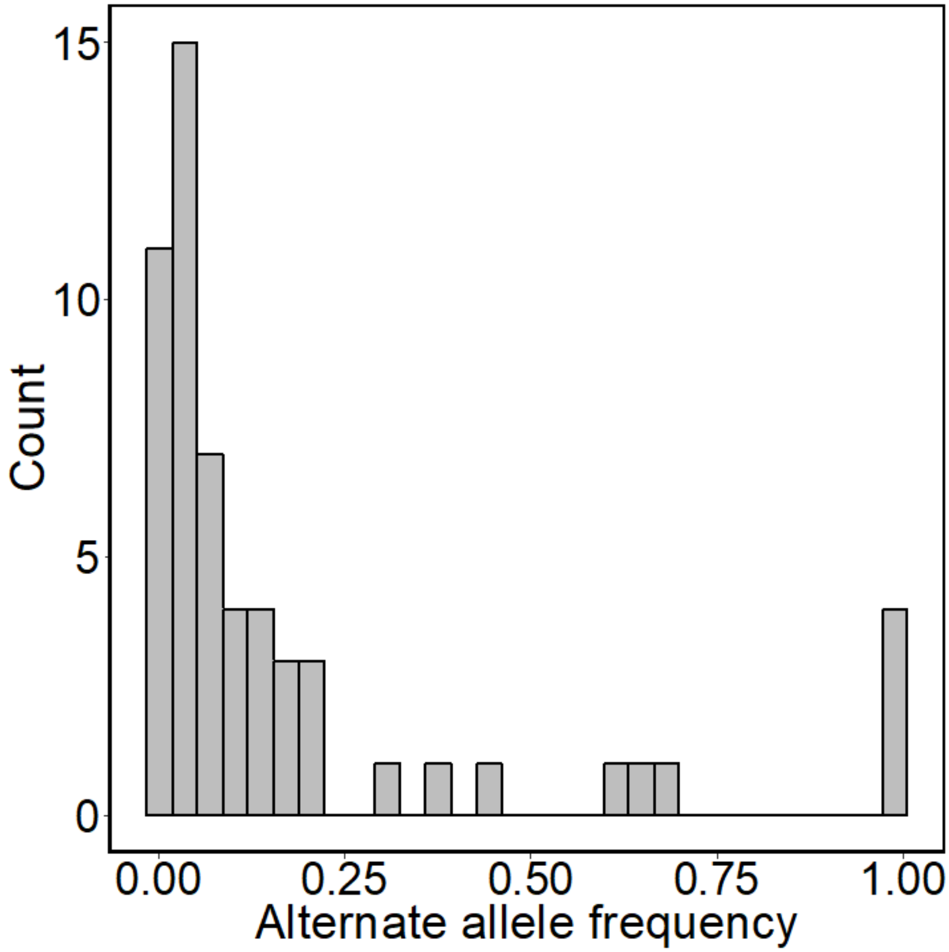

Figure S5. Histogram of the allele frequencies for all mitochondrial base substitutions observed across MA lines from for 9 genotypes of *D. magna*.

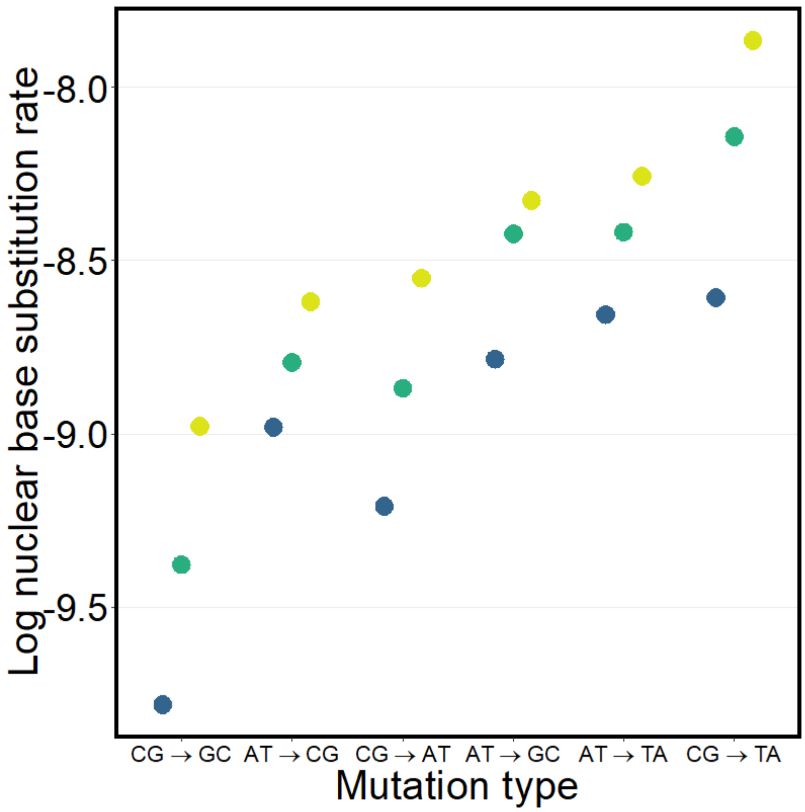

Figure S6. Conditional base substitution rates for each possible type of substitution averaged across MA lines for each population (Finland [yellow], Germany [green] and Israel [blue]).

Supplementary References (including references for data contained in Figure 2)

34. Klüttgen, B., Dülmer, U., Engels, M. & Ratte, H. T. ADaM, an artificial freshwater for the culture of zooplankton. *Water Research* **28**, 743–746 (1994).

35. Crease, T. J. The complete sequence of the mitochondrial genome of *Daphnia pulex* (Cladocera: Crustacea). *Gene* **233**, 89–99 (1999).

36. Colbourne, J. K. *et al.* The ecoresponsive genome of *Daphnia pulex*. *Science* **331**, 555–561 (2011).

37. Langmead, B., & Salzberg, S. L. Fast gapped-read alignment with Bowtie 2. *Nat. Methods* **9**, 357-359 (2012).

38. Bankevich, Anton, Nurk, S., Antipov, D., Gurevich, A. A., Dvorkin, M., Kulikov, A. S., Lesin, V. M, et al. SPAdes: a new genome assembly algorithm and its applications to single-cell sequencing. *J. of Comput. Biol.* **19**, 455-477 (2012).

39. Altschul, S., Gish, W., Myers, E. W. & Lipman, D. J. Basic local alignment search tool. *J. Mol. Biol.* **215**: 403-410 (1990).

40. Pryszcz, L. P., & Gabaldón. T. Redundans: An assembly pipeline for highly heterozygous genomes. *Nucleic Acids Res.* **44**, e113 (2016).

41. Smit, A. F. A, & Hubley, R. RepeatModeler Open-1.0. (http://www.repeatmasker.org) (2008).

42. Smit, A. F. A, Hubley, R. & Green, P. RepeatMasker Open-4.0. (http://www.repeatmasker.org) (2013).

43. McKenna, A., Hanna, M., Banks, E., Sivachenko, A., Cibulskis, K., Kernytsky, A., Garimella, K., et al. The genome analysis toolkit: a MapReduce framework for analyzing next-generation DNA sequencing data. *Genome Res.* **20**, 1297-1303 (2010).

44. Li, H., Handsaker, B., Wysoker, A., Fennell, T., Ruan, J., Homer, N., Marth, G., Abecasis, G., Durbin, R., & 1000 Genome Project Data Processing Subgroup. The sequence alignment/map format and SAMtools. *Bioinformatics* **25**, 2078-2079 (2009).

45. Robinson, J. T, Thorvaldsdottir, H., Winckler, W., Guttman, M., Lander, E. S., Getz, G., and Mesirov, J. P. Integrative genomics viewer. *Correspondence* **29**, 24-26 (2011).

46. Levin, B. A representation for multinomial cumulative distribution functions. *The Annals of Statistics* **9**: 1123-1126 (1981).

47. R Core Team. 2018. R: A language and environment for statistical computing. R Foundation for Statistical Computing, Vienna, Austria. (https://www.R-project.org/).

48. Sung, W., Ackerman, M. S., Gout, J., Miller, S. F., Williams, E., Foster, F. L., & Lynch, M. Asymmetric context-dependent mutation patterns revealed through mutation–accumulation experiments. *Mol. Biol. Evol.* **32**, 1672-1683 (2015).

49. Kumar, S., Stecher, G. & Tamura, K. MEGA7: molecular evolutionary genetic analysis version 7.0 for bigger datasets. Mol. Biol. Ecol. 33, 1870-1874 (2016).

50. Li, H., & Durbin, R. Fast and accurate short read alignment with Burrows-Wheeler transform. *Bioinformatics* **25**, 1754-1760 (2009).

51. Wei, K. H., Grenier, J. K., Barbash, D. A., & Clark, A. G. Correlated variation and population differentiation in satellite DNA abundance among lines of *Drosophila melanogaster*. *P. Natl. Acad. Sci.* **111**, 18793-18798 (2014).

52. Lynch, M., & B. Walsh. Genetics and analysis of quantitative traits. (Sinauer Associates, Sunderland 1998).

53. Schaack, S., Ho, E. K. H. & Macrae, F. Disentangling the intertwined roles of mutation, selection and drift in the mitochondrial genome. Phil. Trans. R. Soc. B. 375, 20190173 (2019).

54. Bull, J. K., Flynn, J. M., Chain, F. J. J. & Cristescu, M. E. Fitness and genomic consequences of chronic exposure to low levels of copper and nickel in *Daphnia pulex* mutation accumulation lines. G3: Genes Genomes Genetics, 9, 61-71 (2019).

55. Xu, S., Schaack, S, Seyfert, A, Choi, E., Lynch, M., & Cristescu, M. E. High mutation rates in the mitochondrial genomes of *Daphnia pulex*. *Mol. Biol. Evol.* **29**, 763-769 (2012).

56. Krasovec, M., Eyre-Walker, A., Sanchez-Ferandin, S. & Piganeau, G. Spontaneous mutation rate in the smallest photosynthetic Eukaryotes. Mol. Biol. Evol. 34, 1770-1779 (2017).

57. Assaf, Z. J., Tilk, S., Park, J., Siegal, M. L., & Petrov, D. A. Deep sequencing of natural and experimental populations of *Drosophila melanogaster* reveals biases in the spectrum of new mutations." *Genome Res.* **27**, 1988-2000 (2017).

58. Denver, D. R., Dolan, P. C., Wilhelm, L. J., Sung, W., Lucas-Lledo, J. L., Howe, P. C., Lewis, S. C., et al. A genome-wide view of *Caenorhabditis elegans* base-substitution mutation processes. *P. Natl. A. Sci.* **106**, 16310-16314 (2009).

59. Denver, D. R., Wilhelm, L. J., Howe, D. K., Gafner, K., Dolan, P. C., & Baer, C. F. Variation in base-substitution mutation in experimental and natural lineages of *Caenorhabditis nematodes*. *Genome Biol. Evol.***4**, 513-22 (2012).

60. Howe, D K., Baer, C. F., & Denver, D. R. High rate of large deletions in *Caenorhabditis briggsae* mitochondrial genome mutation processes. *Genome Biol. Evol.* **2**, 29-38 (2010).

61. Huang, W., Lyman, R. F., Lyman, R. A., Carbone, M. A., Harbison, S. T., Magwire, M. M., & Mackay, T. F. C. Spontaneous mutations and the origin and maintenance of quantitative genetic variation. *ELife* **5**, e14625 (2016).

62. Keightley, P. D., Trivedi, U., Thomson, M, Oliver, F., Kumar, S., & Blaxter, M. L. Analysis of the genome sequences of three *Drosophila melanogaster* spontaneous mutation accumulation lines. *Genome Res.* **19**, 1195-1201 (2009).

63. Konrad, A., Thompson, O., Waterston, R. H., Moerman, D. G., Keightley, P. D., Bergthorsson, U., & Katju, V. Mitochondrial mutation rate, spectrum and heteroplasmy in *Caenorhabditis elegans* spontaneous mutation accumulation lines of differing population size. *Molecular Biology and Evolution* **34**, 1319-1334 (2017).

64. Molnar, R. I., Bartelmes, G., Dinkelacker, I., Witte, H., & Sommer, R. J. Mutation rates and intraspecific divergence of the mitochondrial genome of *Pristionchus pacificus*. *Mol. Biol. Evol.* **28**, 2317-2326 (2011).

65. Schrider, D. R., Houle, D., Lynch, M., & Hahn, M. W. Rates and genomic consequences of spontaneous mutational events in *Drosophila melanogaster*. *Genetics* **194**, 937-954 (2013).

66. Weller, A. M., Rödelsperger, C., Eberhardt, G., Molnar, R. I., & Sommer, R. J. Opposing forces of A/T-biased mutations and G/C-biased gene conversions shape the genome of the nematode *Pristionchus pacificus*. *Genetics* **196**, 1145-1552 (2014).

67. Kumar, S., Stecher, G., Suleski, M., & Hedges, S.B. TimeTree: a resource for timelines, timetrees, and divergence times. *Mol. Biol. Evol.* **34**: 1812-1819 (2017).
